# Association between anthropometry and lifestyle factors and risk of B‐cell lymphoma: An exposome‐wide analysis

**DOI:** 10.1002/ijc.33369

**Published:** 2020-11-12

**Authors:** Fatemeh Saberi Hosnijeh, Delphine Casabonne, Alexandra Nieters, Marta Solans, Sabine Naudin, Pietro Ferrari, James D. Mckay, Yolanda Benavente, Elisabete Weiderpass, Heinz Freisling, Gianluca Severi, Marie‐Christine Boutron Ruault, Caroline Besson, Claudia Agnoli, Giovanna Masala, Carlotta Sacerdote, Rosario Tumino, José María Huerta, Pilar Amiano, Miguel Rodriguez‐Barranco, Catalina Bonet, Aurelio Barricarte, Sofia Christakoudi, Anika Knuppel, Bas Bueno‐de‐Mesquita, Matthias B. Schulze, Rudolf Kaaks, Federico Canzian, Florentin Späth, Mats Jerkeman, Charlotta Rylander, Anne Tjønneland, Anja Olsen, Kristin Benjaminsen Borch, Roel Vermeulen

**Affiliations:** ^1^ Division of Environmental Epidemiology, Institute for Risk Assessment Sciences Utrecht University Utrecht The Netherlands; ^2^ Department of Immunology, Laboratory Medical Immunology, Erasmus MC University Medical Center Rotterdam The Netherlands; ^3^ Unit of Infections and Cancer, Cancer Epidemiology Research Programme, IDIBELL Catalan Institute of Oncology Badalona Spain; ^4^ Centro de Investigación Biomédica en Red: Epidemiología y Salud Pública (CIBERESP) Madrid Spain; ^5^ Institute for Immunodeficiency, Faculty of Medicine and Medical Center University of Freiburg Freiburg Germany; ^6^ Research Group on Statistics, Econometrics and Health (GRECS) University of Girona Girona Spain; ^7^ Nutritional Methodology and Biostatistics Group, International Agency for Research on Cancer World Health Organization Lyon France; ^8^ Section of Genetics International Agency for Research on Cancer Lyon France; ^9^ International Agency for Research on Cancer World Health Organization Lyon France; ^10^ Université Paris‐Saclay, UVSQ CESP U1018 INSERM Villejuif France; ^11^ Gustave Roussy Villejuif France; ^12^ Department of Statistics, Computer Science, Applications “G. Parenti” University of Florence Florence Italy; ^13^ UFR sciences de la santé Université Versailles Saint Quentin en Yvelines, Université Paris‐Saclay, Communaute Paris‐Saclay (Carol) Saint‐Aubin France; ^14^ Versailles Hospital, Unit of Hematology–Oncology Le Chesnay France; ^15^ Epidemiology and Prevention Unit, Fondazione IRCCS Istituto Nazionale dei Tumori Milan Italy; ^16^ Cancer Risk Factors and Life‐Style Epidemiology Unit Institute for Cancer Research, Prevention and Clinical Network—ISPRO Florence Italy; ^17^ Unit of Cancer Epidemiology, Città della Salute e della Scienza University‐Hospital and Center for Cancer Prevention (CPO) Turin Italy; ^18^ Cancer Registry and Histopathology Department Azienda Sanitaria Provinciale Ragusa Italy; ^19^ Department of Epidemiology Murcia Regional Health Council, IMIB‐Arrixaca Murcia Spain; ^20^ CIBER Epidemiología y Salud Pública (CIBERESP) Madrid Spain; ^21^ Public Health Division of Gipuzkoa, BioDonostia Research Institute, San Sebastian; CIBER Epidemiología y Salud Pública Madrid Spain; ^22^ Escuela Andaluza de Salud Pública (EASP) Granada Spain; ^23^ Instituto de Investigación Biosanitaria ibs.GRANADA Granada Spain; ^24^ Centro de Investigación Biomédica en Red de Epidemiología y Salud Pública (CIBERESP) Madrid Spain; ^25^ Unit of Nutrition and Cancer, Catalan Institute of Oncology—ICO, Nutrition and Cancer Group, Bellvitge Biomedical Research Institute—IDIBELL, L'Hospitalet de Llobregat Barcelona Spain; ^26^ Navarra Public Health Institute Pamplona Spain; ^27^ Navarra Institute for Health Research (IdiSNA) Pamplona Spain; ^28^ Department of Epidemiology and Biostatistics Imperial College London London UK; ^29^ MRC Centre for Transplantation King's College London London UK; ^30^ Cancer Epidemiology Unit, Nuffield Department of Population Health University of Oxford Oxford UK; ^31^ Department for Determinants of Chronic Diseases, National Institute for Public Health and the Environment (RIVM) The Netherlands; ^32^ Department of Gastroenterology and Hepatology University Medical Centre Utrecht The Netherlands; ^33^ Department of Molecular Epidemiology German Institute of Human Nutrition Potsdam‐Rehbruecke Nuthetal Germany; ^34^ Institute of Nutritional Sciences University of Potsdam Nuthetal Germany; ^35^ Division of Cancer Epidemiology German Cancer Research Center (DKFZ) Heidelberg Germany; ^36^ Research Group Genomic Epidemiology German Cancer Research Center (DKFZ) Heidelberg Germany; ^37^ Department of Radiation Sciences, Oncology and Cancer center, Department of Hematology Umeå University Umeå Sweden; ^38^ Department of Oncology Lund University Lund Sweden; ^39^ Department of Oncology Skane University Hospital Lund Sweden; ^40^ Department of Public Health University of Copenhagen Copenhagen Denmark; ^41^ Danish Cancer Society Research Center Copenhagen Denmark; ^42^ Department of Community Medicine UiT, The Arctic University of Norway Tromsø Norway; ^43^ Julius Center for Health Sciences and Primary Care University Medical Center Utrecht Utrecht The Netherlands; ^44^ MRC‐PHE Centre for Environment and Health, Department of Epidemiology and Biostatistics Imperial College London London UK

**Keywords:** exposome, exposome‐wide association study, lifestyle, lymphoma, prospective study

## Abstract

To better understand the role of individual and lifestyle factors in human disease, an exposome‐wide association study was performed to investigate within a single‐study anthropometry measures and lifestyle factors previously associated with B‐cell lymphoma (BCL). Within the European Prospective Investigation into Cancer and nutrition study, 2402 incident BCL cases were diagnosed from 475 426 participants that were followed‐up on average 14 years. Standard and penalized Cox regression models as well as principal component analysis (PCA) were used to evaluate 84 exposures in relation to BCL risk. Standard and penalized Cox regression models showed a positive association between anthropometric measures and BCL and multiple myeloma/plasma cell neoplasm (MM). The penalized Cox models additionally showed the association between several exposures from categories of physical activity, smoking status, medical history, socioeconomic position, diet and BCL and/or the subtypes. PCAs confirmed the individual associations but also showed additional observations. The PC5 including anthropometry, was positively associated with BCL, diffuse large B‐cell lymphoma (DLBCL) and MM. There was a significant positive association between consumption of sugar and confectionary (PC11) and follicular lymphoma risk, and an inverse association between fish and shellfish and Vitamin D (PC15) and DLBCL risk. The PC1 including features of the Mediterranean diet and diet with lower inflammatory score showed an inverse association with BCL risk, while the PC7, including dairy, was positively associated with BCL and DLBCL risk. Physical activity (PC10) was positively associated with DLBCL risk among women. This study provided informative insights on the etiology of BCL.

## Abstract 2

**What's new?**

The “exposome” includes all non‐genetic exposures (e.g. diet, viral, environmental, etc.), with the goal of understanding how those exposures may affect an individual's health. In this study, the authors used a technique called “EWAS” (exposome‐wide association study) to identify multiple factors that are associated with B‐cell lymphoma (BCL) risk. Their results confirm both previously reported risk factors and protective factors. In addition, they identify several previously unknown associations. These new insights, gained via the analysis of multiple exposures, suggest that traditional single‐factor approaches may be suboptimal compared with an EWAS approach.

**Abbreviations**
arMED: adapted relative Mediterranean diet score
BCL: B‐cell lymphomas
BMI: body mass index
CLL: chronic lymphocytic leukemia/small lymphocytic lymphoma
DLBCL: diffuse large B‐cell lymphoma
EPIC: the Italian European Prospective Investigation into Cancer and Nutrition cohort
EWAS: exposome‐wide association study
FDR: false discovery rate
FL: follicular lymphoma
IARC: International Agency for Research on Cancer
IL: interleukin
ISD: inflammatory score of the diet
LASSO: the least absolute shrinkage and selection operator technique
MD: Mediterranean diet
METS: Metabolic Equivalent of Task/week
MM: multiple myeloma/plasma cell neoplasm
NHL: non‐Hodgkin lymphoma
PC: principal component
PCA: principal component analysis
SEP: socioeconomic position
WHR: waist to hip ratio

## 1 INTRODUCTION

B‐cell lymphomas (BCLs) are an etiologically, clinically, and histologically heterogeneous group of malignant diseases of B lymphocytes. Immunodeficiency and autoimmunity are strong B‐cell lymphoma risk factors.^1^ Epidemiological studies showed that the risk of BCL is associated with anthropometry measures, lifestyle, viral, environmental and occupational factors (collectively called the exposome).^2^, ^3^, ^4^, ^5^, ^6^, ^7^, ^8^ Moreover, in the last two decades, reports from epidemiological studies suggested differences in risks among BCL subtypes for a wide range of risk factors.^2^

To better understand the role of risk factors in the occurrence of BCL, it would be preferable to study a large set of lifestyle factors (exposome) in a single study. Few methods are available to comprehensively evaluate the role of specific risk factors with disease. Recently, a study design analogous to genome‐wide association studies, the exposome‐wide association study, or equivalently, environment‐wide association study (EWAS), has been proposed to search for and validate exposures associated with complex diseases. Instead of testing one or only a few associations at a time, EWAS evaluates multiple exposures for association, with proper adjustment for multiplicity and collinearity of comparisons. EWAS techniques have recently been used to assess environmental factors in relation to chronic diseases (eg, Type 2 diabetes, high blood pressure and peripheral arterial disease) and mortality.^9^, ^10^, ^11^

In this study, we aimed to use an exposome‐wide approach to evaluate multiple lifestyle exposures and determine both their independent and combined roles (using a multivariable penalized regression algorithm and principal component [PC] approaches) with respect to the risk of BCL and major subtypes using data from the European Prospective Investigation into Nutrition and Cancer cohort (EPIC).

## 2 MATERIALS AND METHODS

The EPIC study is a prospective cohort involving 23 centers from 10 European countries (Denmark, France, Germany, Greece, Holland, Italy, Norway, United Kingdom, Spain and Sweden). The rationale and study design have been described previously.^12^, ^13^ In brief, 521 324 subjects, mostly aged 30 to 70 years, were recruited between 1992 and 2000. Ethical review boards from International Agency for Research on Cancer (IARC) and local participating centers approved the study and all participants gave their written informed consent. Of the 521 324 EPIC cohort participants, we excluded prevalent cancer cases at baseline (n = 25 184), subjects with missing follow‐up information (n = 4148), with incomplete information on diet or lifestyle questionnaires (n = 6259), or those with extreme caloric intake (top and bottom 1% of the total energy intake to energy requirement ratio) (n = 9573) and incident cases of non‐BCL lymphomas (n = 734). This left a cohort of 475 426 subjects, including 2402 incident BCL and 473 024 participants free of cancer.

Validated country‐specific questionnaires were used to collect information on the usual diet during the year before recruitment; namely through self‐administered semi‐quantitative food frequency questionnaires or diet history questionnaires administered through a personal interview, and semi‐quantitative food‐frequency questionnaires combined with a food record.^13^, ^14^ Lifestyle questionnaires were used to obtain information on sociodemographic characteristics, physical activity, medical history and alcohol and tobacco consumption. Anthropometric measures were also ascertained at recruitment.^15^

### 2.1 Follow‐up and outcome assessment

Primary incident lymphoma cancer cases were identified by linkage with national cancer registries in Denmark, Italy, the Netherlands, Norway, Spain, Sweden and the United Kingdom. A combination of methods were used in France, Germany and Greece, including cancer registries, health insurance records, and active follow‐up contacting participants or their next‐of‐kin. Mortality data were retrieved from regional or national mortality registries. The follow‐up period was defined from the age at recruitment to the age at first lymphoma diagnosis, death or last complete follow‐up (December 31, 2013), depending on which occurred first.

Diagnoses of primary incident lymphoma cases were based on the International Classification of Diseases for Oncology, third edition and grouped according to recommendations of the InterLymph Pathology Working Group.^1^ In the current analysis, only mature B‐cell lymphomas (Table S1) were considered, which were further categorized into diffuse large B‐cell lymphoma (DLBCL), follicular lymphoma (FL), chronic lymphocytic leukemia (CLL) (including small lymphocytic leukemia), multiple myeloma/plasma cell neoplasm (MM), and “other” entities (ie, those cases in which the B‐cell lymphoma subtype is unknown or does not fall within the above‐mentioned subtypes).

### 2.2 Exposures assessment per category

*Anthropometry measures* included height, weight, hip circumference, waist circumference, body mass index (BMI, kg/m^2^) and waist to hip ratio. Participants' height, weight, hip circumference and waist circumference were measured at baseline, except for France, Oxford and Norway, where self‐reported measures were obtained via questionnaire.^15^, ^16^, ^17^

*Smoking status* included ever smoking, current smoking, currently smoking cigarettes, currently smoking cigars, duration of smoking, duration of cigarettes smoking, >15/d currently smoked cigarettes and >25/d currently smoked cigarettes.

*Alcohol intake*: Data on alcohol intake were collected through the dietary questionnaire (alcohol intake over 12 months prior to recruitment) and in the lifestyle questionnaire (consumption of alcoholic beverages at different ages in the past) and expressed in grams of ethanol per day (g/d).

*Medical history*: Participants stated whether they had ever had myocardial infarction, stroke, hypertension, diabetes, reported a cardiovascular problem.

*Physical activity*: The assessment of physical activity measures is described in detail elsewhere.^18^, ^19^, ^20^ Current occupational physical activity was based on employment status and on the level of physical activity at current work, which was later coded in categories (sedentary occupation, standing occupation, manual work, heavy manual work and unemployed). Information on housework, do‐it‐yourself work, gardening and climbing stairs was combined to estimate the overall household activities and walking, cycling and sport activities were combined to determine the overall recreational activities. Subsequently, energy expenditure using metabolic equivalent values was calculated, according to the Compendium of Physical Activities.^21^ Sex‐specific total physical activity index, Cambridge physical activity index (1‐4 levels) and IARC physical activity score (1‐3 levels) were also included in current analyses.

*Diet*: The 16 main food groups included were potatoes and other tubers; vegetables; legumes; fruits (including nuts and seeds); dairy products; cereal and cereal products; meat and meat products; fish and shellfish; egg and egg products; fat; sugar and confectionary; cakes and biscuits; nonalcoholic beverages; alcoholic beverages; condiments and sauces; soups and bouillons.

*Nutrient*: Nutrient values of all items from the 24‐hour dietary recalls were standardized to build the EPIC Nutrient Database.^22^ For each nutrient, the nutrient density was calculated by dividing the caloric value of that nutrient by the total caloric intake. For nutrients without caloric value (vitamins, flavonoids, cholesterol, calcium), the weight of the nutrient was divided by the total caloric intake.

*Dietary pattern*: The Mediterranean diet (MD) score was assessed using the adapted relative MD (arMED) score.^23^, ^24^ In brief, the arMED is a 16‐point linear score that incorporates eight key dietary components: six components presumed to reflect the MD [fruit (including nuts and seeds), vegetables, legumes, fish (including seafood), olive oil and cereals] and two components consumed in low quantity in the MD (dairy products and meat). The sum of these points was used to define the MED score, that ranged from 0 to 16 (from the lowest to the highest adherence).^24^ The dietary inflammatory potential was assessed by means of an inflammatory score of the diet (ISD), calculated using 28 dietary components and their corresponding inflammatory weights.^25^, ^26^ Overall, the ISD is a relative index that categorizes individual's diets from maximally anti‐inflammatory (corresponding to lower scores) to maximally pro‐inflammatory (higher values).

*Socioeconomic position* (SEP): Educational level (primary school or less, technical/professional school, secondary school, longer education including university degree) was used as an indirect measure of SEP. A particular advantage of investigating education is avoiding reverse causation bias: diseases may lead to downward occupational mobility and reduced income, but generally will not affect educational status achieved by early adulthood.

### 2.3 Statistical analyses

Table S2 shows the list of 84 exposure variables included in the study. Exposure variables with <25% missing data (Table S3) were imputed based on a maximum likelihood estimation method which was informed by the observed correlation structure within the data. To understand the structure of our data and see which exposures are related to each other, we calculated Spearman rank correlation between each two variables adjusted for age, sex and country. Spearman correlation coefficients were visualized with a heatmap where variables were arranged using a hierarchical clustering algorithm. The larger the correlation between a pair of variables, the closer in proximity they appear in the heatmap. Absolute correlations below 0.2 were omitted and remaining correlations were plotted in a “circus” plot.

Univariate and age‐, sex‐, and country‐adjusted Cox proportional hazards models were used to examine the association between each exposure and BCL and its subtypes. Subsequently, to examine country heterogeneity, we fitted each model per country (age and sex adjusted) and pooled the estimates by conducting a random‐effects meta‐analysis. The coefficient of inconsistency *I* ^2^ was used as a metric to assess heterogeneity between countries, with a *P* value <.05 to be regarded as statistically significant evidence for between country heterogeneity. Cox analyses were stratified by sex and median age at recruitment (≤55, >55 years). Sensitivity analyses were performed by excluding cases with less than 2 years of follow‐up (n = 176 cases) and centers with self‐reported anthropometry data (France, Oxford and Norway) and without comprehensive physical activity data (Norway, Umea).

Given the correlation between exposures, univariate regression analysis is prone to increased false positive results.^27^ Therefore, we used the least absolute shrinkage and selection operator (LASSO) technique, a multivariable penalized regression algorithm,^28^, ^29^ to identify exposures associated with BCL. LASSO technique is a powerful method that performs two main tasks: regularization and feature selection. In order to do so, the method applies a shrinking (regularization) process in which the coefficients of the regression variables are penalized, thus shrinking some of them to zero. During the feature selection process, the variables that still have a nonzero coefficient after shrinkage are selected to be part of the model. Optimal tuning parameter *λ*, which controls the strength of the penalty, was obtained by 5‐fold cross‐validation. All exposures were standardized. Dummy variables were defined for country and together with age and sex were forced into the “regularized” Cox model by decreasing their penalty factor to zero. We also applied an Elastic‐Net approach^29^ that combines the penalties of ridge and LASSO regressions to get the best of both. The method effectively shrinks coefficients (similar to ridge regression) and set some coefficients to zero, like in LASSO. As the results were similar to the LASSO technique, we therefore present the former.

Finally, PC analysis (PCA) was applied to reduce the spectrum of the exposures into a smaller number of clusters of related exposures. Varimax orthogonal rotation method was performed for PCA ensuring absence of redundancy, and component scores were calculated for each individual. Components with eigenvalue >1 were included. age‐, gender‐, country‐adjusted Cox regression was performed on B‐cell lymphoma status for all PCs separately and collectively. Moreover, Cox analyses were stratified by sex and median age at recruitment (≤55, >55 years).

Statistical analyses were performed using the R 3.4.1 language and environment (The R Foundation for Statistical Computing, Vienna, Austria) and IBM SPSS Statistics 25. Statistical *P* values were two sided and were corrected for multiple testing using false discovery rate (FDR) method.

## 3 RESULTS

After an average of 13.94 years follow‐up (SD = 4.03, median = 14.86), 2402 participants developed BCL. Of all BCL cases, 22.4% (n = 537) were diagnosed with CLL, 15.9% (n = 381) with FL, 20.3% (n = 488) with DLBCL, and 28.1% (n = 676) with MM (Table 1). Subjects diagnosed with BCL were more likely to be older, less educated and overweight (higher BMI) at recruitment as compared with the whole population. The percentage of male participants was higher in cases than in the whole population (42.6 vs 29.8, respectively). Distribution of BCL subtypes across countries is shown in Table S4.

**TABLE 1:** General characteristics of study population

	Whole cohort	BCL
Size, n	475 426	2402
Age, mean (SD)	51.2 (9.94)	55.7 (8.10)
Female (%)	70.2	57.4
BMI, mean (SD)	25.4 (4.27)	25.97 (4.24)
Education (%)		
None/primary school	30.3	35.1
Technical/professional school	23.8	27.5
Secondary school	21.0	15.7
Longer education (incl. university degree)	24.9	21.7
Country (%)		
France	14.2	8.5
Italy	9.4	9.1
Spain	8.4	8.1
United Kingdom	15.8	17.7
The Netherlands	7.7	7.2
Greece	5.5	1.6
Germany	10.2	7.1
Sweden	10.2	14.3
Denmark	11.5	21.1
Norway	7.1	5.4
B‐cell lymphoma subtypes (%)		
DLBCL		20.3
CLL		22.4
FL		15.9
MM		28.1
Others		13.3

Abbreviations: BMI, body mass index; CLL, chronic lymphocytic leukemia/small lymphocytic leukemia; DLBCL, diffuse large B‐cell lymphoma; FL, follicular lymphoma; MM, plasma cell neoplasm/multiple myeloma.

#### 3.1 Data correlation structure

Means and medians for exposures in continuous scale and frequencies for exposures in categories are given in Table S2 for the total study population. A large part (87.9%) of absolute correlations between exposures were lower than 0.4 (Figure S1). As expected, correlated exposures were mostly within the same category (Figure S2).

#### 3.2 Standard Cox regression

**FIGURE 1:**
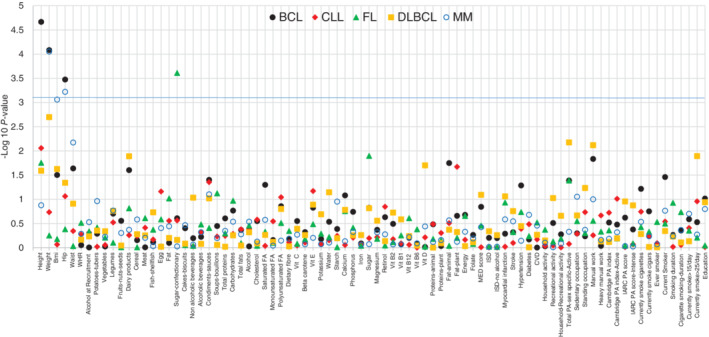
Standard Cox regression for individual exposure *adjusted for country, age* and *sex* in total BCL and subtypes. Vertical axis shows the −Log10 p FDR value and blue line shows pFDR = 0.05. See Table S2 for name, type and unit of the exposures. BCL, B‐cell lymphoma; FDR, false discovery rate [Color figure can be viewed at wileyonlinelibrary.com]

In a first set of analyses, all BCL cases were pooled together and univariable and multivariable Cox regression models adjusted for age, gender and country were performed for each exposure. Results of the univariable models are shown in Table S5. In multivariable models, for BCL, three anthropometry measures reached the significance level after multiple testing correction (Figure 1), namely, height (*β* = .014, *P* value = 2.16E‐05, pFDR = 0.002), weight (*β* = .007, *P* values = 8.26E‐05, pFDR = 0.003) and hip circumference (*β* = .010, *P* values = .0003, pFDR = 0.009).

The analyses for histological subtype did not show any significant associations between the included exposures and CLL and DLBCL (Figure 1). In contrast, we identified two positive associations for MM (Figure 1) including weight (*β* = .012, *P* value = 8.86E‐05, pFDR = 0.007) and hip circumference (*β* = .017, *P* value = 0.0006, pFDR = 0.02). BMI (*β* = .031, *P* value = .0008, pFDR = 0.03) was significantly associated with MM. Moreover, consumption of sugar and confectionary (*β* = .002, *P* value = .0002, pFDR = 0.02) was associated with an increased hazard of FL (Table S6, lower panel).

Next, Cox regression analyses were performed for each country independently and per‐country estimates were pooled (random effect meta‐analysis). Among anthropometry variables, height was associated with increased risk of BCL after multiple testing corrections (Figure 3). We found similar results for MM without significant heterogeneity between countries while the association between consumption of sugar and confectionary and FL risk was no longer statistically significant (*β* = .004, *P* value = .01, *P* heterogeneity = .06) (Figure S3, Table S6, upper panel). In addition, weight (*β* = .013, *P* value = .0006), currently smoking >25/d (*β* = .92, *P* value = .001) and manual work (*β* = .42, *P* value = .002) were positively associated with risk of DLBCL.

The reported associations between BCL, FL, DLBCL and MM and several exposures differed by sex and age (≤55 years, >55 years) (Table S7). The associations between height and weight and BCL, weight and DLBCL and consumption of sugar and confectionary and FL were present among women.

#### 3.3 Penalized Cox regression

LASSO models confirmed the reported findings of the standard Cox model for BCL and the subtypes and selected additional exposures associated to BCL and/or DLBCL (Table 2). Intake of dairy products, total fats with animal origin, physical activity, manual work, currently smoking >25/d and history of myocardial infarction were positively associated with risk of BCL and/or DLBCL while intake of condiments and sauces, polyunsaturated fatty acids, fruit, nuts and seeds, Vitamin D and retinol, history of hypertension and stroke, standing occupation, education, current smoker and currently smoke cigars were negatively associated with risk of BCL and/or DLBCL.

**TABLE 2:** Regression coefficient of the selected exposures by LASSO model for total BCL and major subtypes

		BCL	CLL	FL	DLBCL	MM
Exposure	PC	Estimate	Estimate	Estimate	Estimate	Estimate
Height	16	0.008				
Weight	5	0.001			0.005	0.004
Hip circumference	5	0.004				0.001
Fruit, nuts, and seeds	1	−0.000002				
Dairy products	7	0.0001			0.0002	
Sugar and confectionary	11	0.00002		0.001		
Condiments and sauces	17	−0.001			−0.0001	
Total polyunsaturated fatty acids	6	−0.002				
Total fats, animal origin	2	0.002				
Vitamin D	15				−0.016	
Retinol	19	−0.00002				
Current smoker	3	−0.044				
Currently smoking >25/day	18				0.330	
Currently smoking cigars	19	−0.030				
Total physical activity index (sex‐specific) (Active)	10	0.022			0.141	
Manual work	10	0.082			0.122	
Recreational activity (METS)	8				0.0003	
Standing occupation	20				−0.040	
Hypertension	14	−0.042				
Myocardial infarction	21				0.182	
Stroke	21				−0.010	
Education	16	−0.010				

*Note*: Country, age and gender were forced into the penalized Cox models. All variables were standardized. Penalty parameter, lambda, was derived using 5‐fold cross‐validation. Regression coefficients were obtained at lambda minimum (the value of *λ* at the lowest cross‐validation error). Positive coefficients indicate that an exposure is associated with higher risk of lymphoma, and vice versa for negative coefficients. See Table S2 for name, type and unit of the exposures.

Abbreviations: BCL, B‐cell lymphoma; CLL, chronic lymphocytic leukemia/small lymphocytic leukemia; DLBCL, diffuse large B‐cell lymphoma; FL, follicular lymphoma; METS, Metabolic Equivalent of Task/week; MM, plasma cell neoplasm/multiple myeloma; PC, principal component.

#### 3.4 Principal component analysis

**FIGURE 2:**
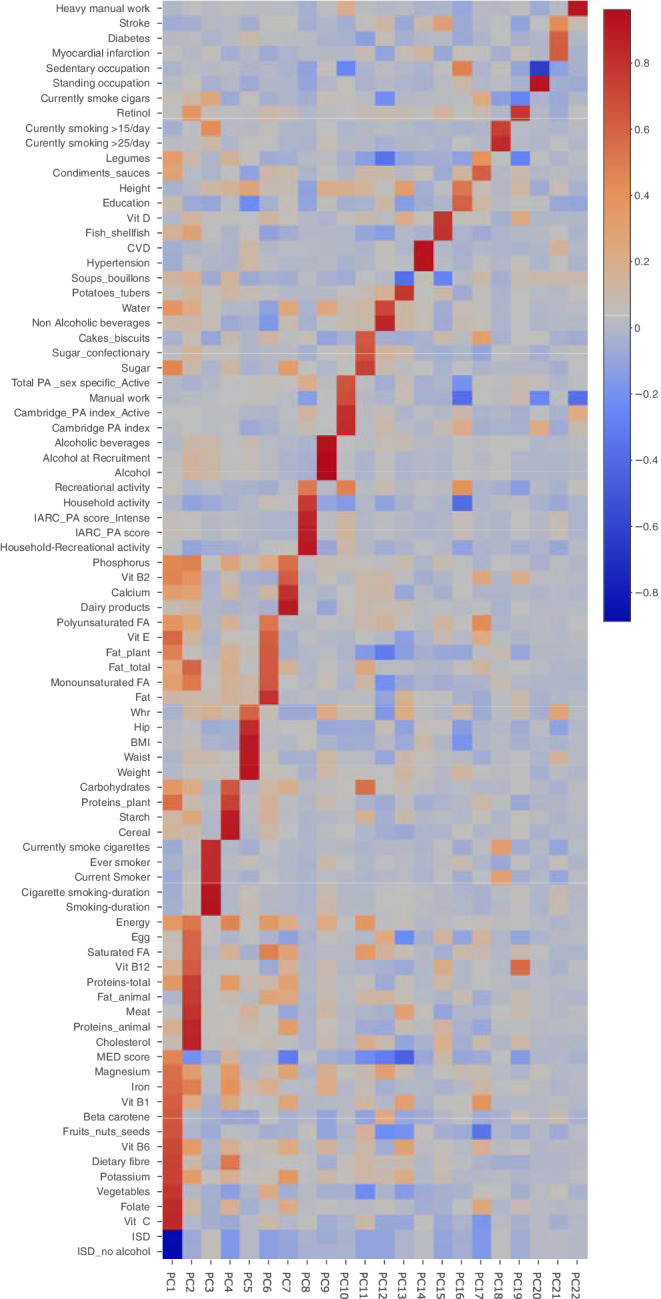
A heatmap for the correlation of each exposure with each principal component. The color of the intersection of an exposure (vertical axis) and a principal component (horizontal axis) indicates the dimension of the correlation: dark blue indicates a highly negative correlation; dark red indicates a highly positive correlation; The staircase‐like high correlation line in the figure indicates that all exposures are correlated with one of the principal components without much overlap. See Table S2 for name, type and unit of the exposures [Color figure can be viewed at wileyonlinelibrary.com]

To investigate how the exposures might have a combined effect, we used PCA. The exposures in total population were merged into 22 PCs with eigenvalue>1 explaining 78.5% of the data variation (Figure 2, Table S8). As shown in Figure 2, all exposures were correlated with one of the PCs without much overlap. In multivariable Cox models adjusted for age, sex and country, we found six PCs (PC5, PC7, PC10, PC11, PC12 and PC15) reached to significance of 0.05 in relation to BCL and/or subtypes, among which PC5 including anthropometry measures was significant after multiple testing correction (Table S9). Multivariable Cox models mutually adjusted for other PCs essentially showed the same PCs, except for PC12, which was characterized by high water and nonalcoholic beverage consumption and no longer associated with MM after mutual adjustment for other PCs (Table 3). Moreover, we found a lower risk of BCL related to PC1, which includes certain dietary features of the MD and diet with lower inflammatory score. PC15 including consumption of fish, shellfish and Vitamin D showed a protective effect on DLBCL while consumption of dairy products, calcium and phosphorus (PC7) were related to increased risk of BCL and DLBCL. The PC10 involving physical activity variables was associated with increased risk of BCL and DLBCL. Higher consumption of sugar, confectionery and carbohydrate products (PC11) increased FL risk. Majority of the selected exposures by LASSO were among those present in the significant PCs. This indicates that these individual exposures were actually part of a broader construct and should be interpreted in that context.

**TABLE 3:** Multivariable HRs and 95% CIs of the PCs for total BCL and major subtypes mutually adjusted for other PCs

	BCL		CLL		FL		DLBCL		MM	
PC	HR (95% CI)	*P*	HR (95% CI)	*P*	HR (95% CI)	*P*	HR (95% CI)	*P*	HR (95% CI)	*P*
1	0.94 (0.89‐0.99)	.03	0.94 (0.84‐1.05)	.26	0.96 (0.84‐1.10)	.58	0.97 (0.87‐1.10)	.66	0.91 (0.82‐1.01)	.08
2	1.03 (0.98‐1.08)	.27	1.10 (0.99‐1.21)	.07	0.97 (0.85‐1.10)	.63	0.98 (0.88‐1.10)	.79	1.01 (0.92‐1.10)	.91
3	0.99 (0.95‐1.03)	.65	0.99 (0.90‐1.08)	.75	0.94 (0.85‐1.05)	.31	1.01 (0.92‐1.11)	.78	0.99 (0.91‐1.07)	.82
4	1.03 (0.99‐1.08)	.17	1.00 (0.91‐1.11)	.95	1.02 (0.91‐1.15)	.73	0.99 (0.90‐1.11)	.94	1.06 (0.98‐1.16)	.16
5	1.09 (1.04‐1.15)	.0001	1.06 (0.96‐1.18)	.25	1.02 (0.89‐1.16)	.78	1.15 (1.03‐1.28)	.01	1.18 (1.07‐1.29)	.0001
6	1.00 (0.95‐1.05)	.97	0.91 (0.81‐1.02)	.10	0.94 (0.82‐1.08)	.42	0.96 (0.85‐1.08)	.48	1.06 (0.96‐1.16)	.25
7	1.04 (1.00‐1.09)	.04	1.01 (0.92–1.10)	.89	1.09 (0.98‐1.20)	.11	1.12 (1.03‐1.22)	.01	1.02 (0.94‐1.10)	.62
8	1.00 (0.96‐1.05)	.90	0.98 (0.89‐1.07)	.61	0.95 (0.85‐1.06)	.39	1.05 (0.95‐1.15)	.33	1.00 (0.92‐1.08)	.95
9	1.03 (0.99‐1.07)	.20	1.04 (0.96‐1.14)	.31	1.08 (0.97‐1.20)	.15	1.02 (0.93‐1.12)	.67	0.98 (0.90‐1.06)	.62
10	1.06 (1.02‐1.11)	.01	1.07 (0.98‐1.17)	.14	1.10 (0.98‐1.22)	.10	1.12 (1.02‐1.22)	.02	1.07 (0.98–1.16)	.13
11	1.04 (0.99–1.08)	.07	0.98 (0.90‐1.08)	.74	1.13 (1.03‐1.25)	.01	1.06 (0.97‐1.16)	.18	1.02 (0.94–1.10)	.67
12	1.05 (0.99‐1.13)	.13	1.06 (0.92‐1.22)	.43	1.02 (0.86‐1.21)	.82	1.09 (0.94‐1.26)	.25	1.09 (0.95‐1.23)	.21
13	1.04 (0.98‐1.10)	.19	1.01 (0.89‐1.15)	.86	1.02 (0.87‐1.18)	.82	1.12 (0.98‐1.27)	.08	1.07 (0.96‐1.19)	.21
14	0.98 (0.94‐1.02)	.24	0.98 (0.90‐1.07)	.64	0.92 (0.82‐1.03)	.16	0.99 (0.90‐1.09)	.81	1.03 (0.95‐1.11)	.50
15	0.99 (0.95–1.05)	.86	1.04 (0.94‐1.16)	.45	1.06 (0.94‐1.20)	.35	0.86 (0.76‐0.98)	.02	1.05 (0.96–1.16)	.27
16	1.02 (0.97‐1.08)	.37	1.05 (0.94‐1.17)	.37	1.08 (0.94‐1.23)	.28	1.01 (0.90‐1.14)	.81	0.97 (0.88‐1.07)	.59
17	0.95 (0.90‐1.00)	.06	0.91 (0.81‐1.03)	.12	0.95 (0.82‐1.09)	.47	1.02 (0.91–1.15)	.69	0.91 (0.82‐1.02)	.10
18	0.99 (0.95‐1.04)	.83	1.01 (0.92–1.11)	.83	1.00 (0.89‐1.13)	.96	1.05 (0.96‐1.15)	.28	0.95 (0.86‐1.04)	.25
19	1.00 (0.96–1.05)	.99	0.96 (0.88‐1.06)	.46	1.03 (0.92‐1.16)	.56	1.02 (0.92‐1.12)	.76	1.00 (0.92‐1.09)	.97
20	0.98 (0.94–1.02)	.37	0.94 (0.85‐1.03)	.17	0.97 (0.87‐1.08)	.61	0.92 (0.83‐1.02)	.11	1.03 (0.95‐1.13)	.43
21	1.01 (0.97‐1.05)	.53	1.03 (0.95–1.11)	.47	0.91 (0.80‐1.03)	.15	1.05 (0.97‐1.14)	.24	1.02 (0.95‐1.10)	.57
22	0.98 (0.94‐1.02)	.29	1.03 (0.96‐1.11)	.40	0.94 (0.84‐1.04)	.23	0.95 (0.87‐1.03)	.22	0.99 (0.92‐1.06)	.76

*Note*: Model included age, gender, country and all PCs; Figure 2 and Table S8 shows name and loading of individual variables in PCs.

Abbreviations: BCL, B‐cell lymphoma; CIs, confidence intervals; CLL, chronic lymphocytic leukemia/small lymphocytic leukemia; DLBCL, diffuse large B‐cell lymphoma; FL, follicular lymphoma; HR, hazard ratios; MM, plasma cell neoplasm/multiple myeloma; PC, principal component.

The stratified analyses revealed that the associations between BCL and the subtypes and certain PCs slightly differ between males and females (Table 4) and by age (Table S10). These analyses showed a positive association between PC5, PC7, PC10, PC11, PC12 and PC13 and DLBCL and between PC9, PC11, PC13 and FL among women.

**TABLE 4:** Multivariate Cox regression of the principal components for total BCL and major subtypes *mutually adjusted for other PCs stratified by sex*

HR (95% CI)										
PC	BCL		CLL		FL		DLBCL		MM	
	Male	Female	Male	Female	Male	Female	Male	Female	Male	Female
1	0.94 (0.87–1.03)	0.94 (0.88‐1.01)	0.99 (0.84‐1.17)	0.91 (0.78‐1.07)	0.98 (0.77‐1.24)	0.95 (0.81‐1.12)	1.01 (0.84‐1.21)	0.92 (0.79‐1.07)	**0.83 (0.71‐0.98)**	0.98 (0.86‐1.12)
2	1.03 (0.96‐1.10)	1.03 (0.96‐1.11)	1.11 (0.97‐1.27)	1.09 (0.93‐1.28)	0.84 (0.68‐1.04)	1.06 (0.90‐1.25)	1.03 (0.89‐1.21)	0.92 (0.79‐1.08)	1.04 (0.92‐1.18)	0.95 (0.83‐1.10)
3	1.03 (0.97‐1.10)	0.95 (0.90‐1.01)	1.06 (0.93‐1.20)	0.91 (0.79‐1.04)	0.99 (0.82‐1.19)	0.93 (0.81‐1.07)	1.10 (0.95‐1.27)	0.93 (0.82‐1.06)	1.05 (0.94‐1.18)	0.92 (0.81‐1.03)
4	1.03 (0.96–1.10)	1.02 (0.96‐1.09)	0.97 (0.84‐1.11)	1.03 (0.89‐1.19)	1.07 (0.89‐1.29)	0.96 (0.82‐1.12)	1.00 (0.85‐1.17)	1.00 (0.87‐1.15)	1.04 (0.92‐1.18)	1.07 (0.94‐1.20)
5	**1.09 (1.01‐1.18)**	**1.10 (1.03‐1.17)**	1.07 (0.91‐1.25)	1.07 (0.92‐1.23)	0.97 (0.77‐1.23)	1.06 (0.91‐1.24)	0.97 (0.81‐1.17)	**1.25 (1.09‐1.43)**	**1.29 (1.12‐1.48)**	1.09 (0.97‐1.23)
6	1.02 (0.95‐1.09)	0.97 (0.90‐1.05)	0.93 (0.80‐1.08)	0.87 (0.73‐1.04)	1.02 (0.84‐1.26)	0.90 (0.75‐1.08)	1.00 (0.85‐1.19)	0.91 (0.77‐1.08)	1.03 (0.90‐1.17)	1.08 (0.94‐1.24)
7	1.02 (0.96‐1.09)	**1.06 (1.01‐1.13)**	1.02 (0.91‐1.15)	0.99 (0.87‐1.13)	1.12 (0.96‐1.32)	1.05 (0.92‐1.20)	1.00 (0.88‐1.15)	**1.22 (1.09‐1.37)**	0.99 (0.88‐1.11)	1.05 (0.94‐1.18)
8	1.02 (0.95–1.09)	1.00 (0.94‐1.05)	0.97 (0.85‐1.12)	0.97 (0.86‐1.10)	1.07 (0.87‐1.31)	0.91 (0.80‐1.04)	1.14 (0.97‐1.33)	1.00 (0.89‐1.13)	0.94 (0.82‐1.07)	1.06 (0.95‐1.17)
9	1.01 (0.96‐1.07)	1.05 (0.96‐1.14)	1.03 (0.93‐1.14)	1.10 (0.92‐1.32)	0.98 (0.84‐1.14)	**1.24 (1.04–1.46)**	1.01 (0.90‐1.13)	1.00 (0.82‐1.21)	0.99 (0.90‐1.09)	0.92 (0.77‐1.11)
10	1.06 (1.00‐1.13)	1.06 (1.00‐1.13)	1.10 (0.97‐1.25)	1.04 (0.90‐1.19)	1.03 (0.86‐1.23)	1.14 (0.99‐1.31)	1.03 (0.89‐1.19)	**1.17 (1.03‐1.33)**	1.10 (0.98‐1.24)	1.01 (0.90‐1.15)
11	1.02 (0.96‐1.07)	**1.07 (1.01‐1.14)**	0.98 (0.87–1.10)	0.98 (0.84‐1.14)	1.06 (0.90‐1.25)	**1.22 (1.07‐1.39)**	0.98 (0.88‐1.11)	**1.16 (1.02‐1.32)**	1.05 (0.95‐1.16)	0.94 (0.83‐1.07)
12	1.06 (0.96‐1.18)	1.05 (0.95‐1.15)	1.09 (0.89‐1.33)	1.02 (0.83‐1.25)	0.91 (0.67‐1.25)	1.08 (0.87‐1.34)	0.93 (0.74‐1.18)	**1.25 (1.03‐1.52)**	1.20 (0.99‐1.44)	0.97 (0.81‐1.16)
13	0.98 (0.91‐1.07)	**1.11 (1.02‐1.20)**	1.02 (0.87‐1.21)	0.99 (0.92–1.20)	0.80 (0.62‐1.04)	**1.22 (1.01‐1.47)**	0.89 (0.73‐1.08)	**1.36 (1.15‐1.62)**	1.15 (1.00‐1.33)	0.98 (0.83‐1.16)
14	0.97 (0.91‐1.04)	0.97 (0.92‐1.03)	0.95 (0.83‐1.08)	1.01 (0.89‐1.14)	0.91 (0.75‐1.10)	0.93 (0.81‐1.07)	0.96 (0.83‐1.11)	1.01 (0.90‐1.14)	1.03 (0.92‐1.15)	1.02 (0.92‐1.13)
15	0.99 (0.91‐1.07)	1.00 (0.94‐1.08)	1.01 (0.87‐1.18)	1.07 (0.93‐1.24)	1.11 (0.89‐1.38)	1.04 (0.90‐1.21)	**0.73 (0.59‐0.89)**	0.96 (0.82‐1.13)	1.14 (1.00‐1.29)	1.00 (0.87‐1.15)
16	0.98 (0.90‐1.06)	1.06 (0.99‐1.15)	1.00 (0.86‐1.17)	1.13 (0.96‐1.33)	1.08 (0.86‐1.35)	1.01 (0.85‐1.20)	0.91 (0.77‐1.09)	1.13 (0.96‐1.32)	0.97 (0.84‐1.12)	0.99 (0.85‐1.14)
17	**0.88 (0.81‐0.96)**	1.02 (0.94‐1.11)	0.85 (0.72‐1.01)	0.99 (0.82‐1.18)	0.95 (0.76‐1.18)	0.95 (0.79‐1.14)	0.85 (0.71‐1.02)	1.16 (0.99‐1.37)	0.85 (0.73‐1.00)	1.00 (0.86‐1.16)
18	1.02 (0.96‐1.08)	0.95 (0.88‐1.03)	1.03 (0.93‐1.15)	0.96 (0.80‐1.15)	1.00 (0.85‐1.18)	1.01 (0.85‐1.20)	1.10 (0.98‐1.22)	0.98 (0.83‐1.15)	0.98 (0.88‐1.09)	0.83 (0.68‐1.02)
19	0.97 (0.91‐1.04)	1.02 (0.96‐1.09)	0.95 (0.84‐1.08)	0.96 (0.83‐1.12)	1.08 (0.96‐1.20)	0.97 (0.83‐1.15)	0.93 (0.81‐1.08)	1.13 (0.98‐1.30)	1.01 (0.90‐1.14)	0.96 (0.84‐1.10)
20	1.03 (0.96‐1.10)	**0.94 (0.88‐0.99)**	0.98 (0.85‐1.12)	0.89 (0.78‐1.02)	0.92 (0.76‐1.11)	1.00 (0.87‐1.14)	1.10 (0.95‐1.27)	**0.79 (0.68‐0.91)**	1.01 (0.89‐1.14)	1.07 (0.94‐1.19)
21	1.01 (0.95‐1.06)	1.02 (0.96‐1.08)	1.02 (0.92‐1.13)	1.02 (0.90‐1.16)	**0.72 (0.55‐0.96)**	1.02 (0.88‐1.17)	1.05 (0.94‐1.17)	1.05 (0.93‐1.19)	1.02 (0.92‐1.12)	1.02 (0.92‐1.14)
22	0.97 (0.93‐1.02)	0.99 (0.93‐1.06)	0.98 (0.89‐1.08)	1.12 (1.00‐1.26)	0.91 (0.77‐1.06)	0.98 (0.84‐1.13)	0.98 (0.88‐1.09)	0.90 (0.77‐1.05)	1.01 (0.93‐1.10)	0.90 (0.77‐1.06)

*Note*: Model included age, country and all PCs. Bold values are significant at *P* < .05.

Abbreviations: BCL, B‐cell lymphoma; CI, confidence interval; CLL, chronic lymphocytic leukemia/small lymphocytic leukemia; DLBCL, diffuse large B‐cell lymphoma; FL, follicular lymphoma; HR, hazard ratios; MM, plasma cell neoplasm/multiple myeloma; PC, principal component.

Exclusion of cases diagnosed in the first 2 years of follow‐up (n = 176) did not materially alter the estimates for individual exposures and PCs. Moreover, sensitivity analyses excluding centers with self‐reported anthropometry data and centers without comprehensive physical activity data (n = 178 106) did not change the reported association between anthropometry and BCL and MM, and between physical activity and BCL and DLBCL (data not shown).

## 4 DISCUSSION

In this large prospective cohort study, several anthropometric measures and lifestyle factors were associated with BCL and/or subtypes, with strong evidence for a positive association of anthropometric measures.

In our study, we used a new exposome‐based approach to find risk factors of BCL. It consisted of four analytical parts: investigating the data structure, standard and penalized Cox regression methods to find exposures robustly associated to the risk of BCL, and a PCA to see how the exposures are related. A multivariable penalized regression analysis, like LASSO, is preferable over a regression model considering each individual exposure variable separately, since LASSO has been previously shown to have a more favorable tradeoff between sensitivity and the false discovery proportion in exposome data.^27^, ^30^ With LASSO, a model is created where the effect of each exposure is adjusted for the effect of other associated exposures, while penalizing the nonassociated exposures to null. It has been shown that in the presence of correlated influential variables, LASSO will select at most one of them.^30^ Associated exposures are penalized to zero or even show a reverse association. Therefore, we also performed PCA to investigate which exposures are correlated and have potentially a combined effect.

Several anthropometry variables reflecting obesity (ie, weight, hip circumference, and BMI) as well as height were positively associated with BCL and/or subtypes in our study and previous investigations.^2^, ^31^, ^32^, ^33^ There are several potential mechanisms whereby obesity may increase the risk of BCL. Impaired immune function, chronic inflammatory response, effects on cell proliferation and changes in the metabolism of endogenous hormones, leading to a distortion in the normal balance between cell proliferation, differentiation and apoptosis are some of the postulated putative biological mechanisms.^2^, ^31^, ^33^ Height per se does not cause cancer, but could act as a marker for genotype or environmental exposures that could influence immune system function.^33^, ^34^ Height is a proxy for cell division and total cell reservoir. Larger bodies contain more cells than smaller bodies and therefore have a greater chance that a cell will undergo malignant transformation, escape the body's cancer defense mechanisms, and progress to cancer.^33^ Moreover, height might be related to cancer risk through increased cell turnover mediated by growth factors.^33^

Based on epidemiologic reports, there is growing evidence that diet plays a role in lymphomagenesis although the data for BCL subtypes are scarce. This study provides more support for the role of nutrition in lymphoma. In our study, consumption of sugar, confectionary, and carbohydrates products was associated with increased risk of FL, particularly among female participants. The majority of previous prospective and case‐controls studies on total carbohydrates or the main food sources of carbohydrates and lymphoma risk were null except a positive association reported between high consumption of white bread or pasta and non‐Hodgkin lymphoma (NHL).^35^ Although sugar intake was not associated with NHL,^36^ in a follow‐up study, women who frequently consumed cakes or pies were associated with an elevated risk for NHL.^37^ Studying dietary glycemic index and glycemic load in further studies are warranted to clarify this association.^38^

We found that consumption of dairy products, calcium, riboflavin (B2) and phosphorus may increase the risk of BCL and DLBCL, in particular among females. Previous studies suggested a positive association between dairy products and risk of NHL,^39^, ^40^ particularly for DLBCL.^41^ Milk is a source of fat and protein, which are both thought to be risk factors for NHL,^39^ as well as calcium, riboflavin and Vitamin A.^35^ The positive association between dairy consumption and risk of BCL may be attributed to the effects of dietary calcium and phosphorus, largely found in dairy products, which decrease levels of 1,25(OH)2 Vitamin D [1,25(OH)2D]. 1,25(OH)2 D, the physiologically active form of Vitamin D, is considered an anticarcinogen because it promotes differentiation and apoptosis and inhibits cell growth in preneoplastic and neoplastic cells. Moreover, association between Vitamin D3 and autophagy and activation of antibacterial peptides have been reported.^42^, ^43^ Although a recent pooled study did not support the hypothesis that elevated circulating Vitamin D [25(OH)D] concentration is associated with a reduced risk of NHL,^44^ another study suggested that 25(OH)D insufficiency was associated with inferior survival in DLBCL and T‐cell lymphoma.^45^ Another mechanism involves presence of organochlorines such as dioxins and polychlorinated biphenyls in dairy fat, which are well‐known human carcinogens and immunotoxins and can alter normal B‐cell responses. Finally, bovine leukemia virus associated with lymphosarcoma in cattle may be transmitted through milk to humans, although there is no clear evidence of human infection.

Our study also suggested a possible inverse association between consumption of fish, shellfish and Vitamin D and the risk of DLBCL. Previous reports on fish consumption and risk of lymphoma have yielded inconsistent findings. Possible reasons for discrepancies across studies may reflect varying levels of organochlorine pesticides and polychlorinated biphenyls compounds that have been associated with increased risk of NHL. Thus, adverse health effects related to their high content in some fish may diminish the otherwise protective effects conferred by fish consumption.^39^

We found a possible association between the consumption of animal fats (positive association) and polyunsaturated fatty acids (inverse association) and risk of BCL. Many studies suggest that high‐fat diets are linked to the etiology of NHL. A recent meta‐analysis showed a significant association between total fat consumption and increased risk of NHL and DLBCL, but not for CLL and FL.^46^ They found that only high animal fat consumption increases the risk for NHL with no association with vegetable fat consumption.^46^ A more recent large prospective study, reported increased risk of NHL associated with intakes of total, animal, saturated and trans fat with 14 years of follow‐up. However, these associations did not persist with longer follow‐up.^47^ Animal fats are comprised of saturated fatty acids and unsaturated fats, whereas vegetable fat has a higher concentration of unsaturated fatty acids. A diet high in polyunsaturated fatty acids has been shown to reduce the levels of pro‐inflammatory markers such as interleukin (IL)‐6, IL‐1 receptor antagonist, tumor necrosis factor, and C‐reactive protein, as well as increased levels of anti‐inflammatory factors, such as IL‐10 and transforming growth factor.^48^ On the other hand, saturated fats can modulate immune function by enhancing nuclear factor‐κB activation and antiapoptotic behavior in T cells, in addition to increasing expression of pro‐inflammatory agents such as IL‐6, cyclooxygenase‐2 and inducible nitric oxide synthase.^39^ A few studies suggest that the link might be related to changes in serum levels of leptin and adiponectin that stimulate proliferation and inhibit apoptosis through PI3K/AKT activation.^46^

We previously showed that BCL risk was associated with a higher ISD and a lower adherence to MD,^24^, ^26^ which was confirmed in the present study. The role of inflammation, immune dysregulation and autoimmunity are known in the pathogenesis of lymphoma.^49^ Recent studies further support the inflammatory potential of diet^50^; in particular, for lymphoid neoplasms, positive associations have been recently reported between a pro‐inflammatory dietary score and NHL.^51^ The MD, one of the healthiest traditional dietary patterns, is a plant‐based pattern, where vegetables, fruits, cereals (preferably as whole grain), legumes and nuts are consumed in high amount and frequency. It provides a diet rich in flavonoids, carotenoids and Vitamin C or E, whose important antioxidant properties can neutralize free radicals or prevent DNA damage.^52^, ^53^

Moreover, our study suggests a possible beneficial effect of condiments and sauces intake on BCL and DLBCL. Spices, condiments and sauces cover a broad range of substances with different characteristics. Research indicates that some herbs and spices, or their bioactive components, may act alone or in concert to reduce cancer risk through their antimicrobial, antioxidant and antitumorigenic properties, as well as their direct suppressive effect on carcinogen bioactivation. Nevertheless, the evidence to date with herbs and spices is inconsistent and warrants greater attention.^54^

We found that physical activity (PC10) was positively associated with BCL and DLBCL. Sex stratified analyses showed that the association with DLBCL was limited to the female participants. It should be noted that women reported lower levels of physical activity (in general) and more household activity compared with men in our study. However, it is not clear how physical activity positively influences DLBCL risk. Considering the increased risk of DLBCL for manual work among women (Table S7), we cannot exclude bias due to some unknown harmful co‐exposures such as household pollutants during physical activities to female participants. Our Cox regression models also suggested a positive association between recreational physical activity and DLBCL risk. Exposure to environmental pollutants especially during outdoor recreational physical activity (ie, passive exposure to volatile organic compounds and other chemicals) also cannot be excluded. The results of previous prospective investigations are inconsistent, probably due to the lack of reliable, valid and comprehensive measures of physical activity.^55^ Despite of the relative validity and reproducibility of the physical activity questions across the countries in the EPIC study,^20^ some degree of measurement error and misclassification is likely. Further studies that also incorporate environmental and occupational exposures are needed.

Our study also suggests that heavy smoking (>25 per day) may increase the risk of DLBCL. A pooled analysis of case‐control studies within the InterLymph consortium showed that current smoking was associated with a significant 30% increased risk of FL, but not NHL overall or other NHL subtypes.^56^ Moreover, a meta‐analysis of seven prospective studies^57^ did not show association between cigarette smoking and NHL. Several factors may explain these inconsistencies including methodological differences in the studies (ie, study design, data collection, categorizations and residual confounding) and differences in population in terms of ethnicity, socioeconomic status, and disease prevalence. Therefore, further research to pursue the association is warranted. One promising direction for future investigation includes refining our understanding of the carcinogens in cigarette smoke and their biological effects that could plausibly contribute to lymphomagenesis.^58^

An inverse association between alcohol intake and BCL has been consistently observed in both large case‐control^2^ and prospective studies.^59^ However, it has been hypothesized that this association is driven by unknown confounders. Unlike previous large observational studies, our study did not show the protective effect of alcohol intake in BCL. In contrast, our findings suggested a positive association between alcohol intake (PC9) and FL among women after adjustment for other PCs (HR = 1.24, 95% CI = 1.04‐1.46) (Table 4). Due to insufficient power of the stratified analyses, this finding should be interpreted with caution and further prospective studies are deemed necessary.

The LASSO analyses showed an inverse association between level of education and risk of BCL. Limited and contradictory literature is published about educational level or other SEP indicators and lymphoma risk.^60^, ^61^ In line with our findings the InterLymph study showed lower risk of lymphoma and DLBCL among highly educated people.^2^ Populations with low SEP may be more exposed to hazard occupational exposures, air pollution, smoking and infections which can increase the risk of lymphoma.

Penalized Cox models revealed an inverse association between history of hypertension and BCL. We recently reported also a negative association with hypertension and with both, systolic and diastolic blood pressure levels in EPIC for all‐type lymphomas and for the subgroup of NHL.^62^ Inflammatory processes play an important role in the pathogenesis of hypertension. Different subpopulations of immune cells involved in innate and adaptive immune responses are involved in inflammatory processes and exert their effects in part via production of various pro‐inflammatory and anti‐inflammatory cytokines,^63^ so there may be some mechanistic explanation, but this would need a more detailed investigation.

The strengths of this study include its prospective design, long follow‐up, and large size which allowed us to carry out analyses by BCL subentities. Limitations of our study should be considered when interpreting the results, including potential measurement errors derived from questionnaires, which could lead to systematic and random errors. We cannot rule out that they have affected risk estimates. Our study lacked data on other exposures such as medication, occupational exposures, family history of hematological cancers and medical history of immunologic disorders and viral infections which are among the most important risk factors. In addition, we were unable to take into account any possible changes in dietary and lifestyle habits over time. Although reverse causation could be induced by changes of lifestyle behaviors before recruitment because of early symptoms, after exclusion of the first 2 years of follow‐up, associations were unchanged, possibly indicating a minor role of reverse causation. Furthermore, despite the large number of observed incident cases of BCL, this study might not have sufficient power to detect significant associations within the BCL subtypes with a lower number of cases, in particular in the sex and age stratified analyses. Finally, despite multiple‐testing corrections, we cannot exclude chance findings.

In conclusion, our systematic evaluation confirmed several previously reported risk factors (anthropometric measures, animal fat, dairy and sugar intake) as well as protective factors (MD, diet with lower inflammatory score, fish and Vitamin D, SEP) of BCL and/or subtypes. While our study did not support the previously reported protective effect of alcohol intake on BCL, it revealed several unknown associations (increased risk of DLBCL with smoking and a beneficial effect of condiments and sauces intake for BCL and DLBCL). In this study, we applied a comprehensive approach for conceptualizing the roles and relationships of multiple exposures in the etiology of BCL and could generate some new insight in BCL risk factors. This highlights that traditional approaches of testing single association at a time could be suboptimal compared with a EWAS approach.

## CONFLICT OF INTERESTS

The authors declare no potential conflict of interest. Where authors are identified as personnel of the International Agency for Research on Cancer/WHO, the authors alone are responsible for the views expressed in this article and they do not necessarily represent the decisions, policy or views of the International Agency for Research on Cancer/WHO.

5

## ETHICS STATEMENT

Ethical review boards from IARC and local participating centers approved the study. Written informed consent was obtained from all individual participants included in the study.

## Supporting information


**TABLE S1** ICD‐O‐3 morphology codes
**TABLE S2**. List of included exposures; mean and median for continuous exposures and frequency (%) of categorical exposures in total study population
**TABLE S3**. Missing rate
**TABLE S4**. Case number of BCL and major subtypes stratified by country
**TABLE S5**. Univariate Cox model for individual exposure in total BCL and subtypes
**TABLE S6**. Estimates and SE of the significant associated exposures from meta‐analysis of per‐country Cox model coefficients adjusted for age and gender (upper part) and estimates and SE of the significant associated exposures of Cox model adjusted for country, age, and sex (lower part)
**TABLE S9**. HRs and 95%CI of the significant principal components for total BCL and major subtypes adjusted for age, gender, and country
**TABLE S10**. Multivariate Cox regression of the principal components for total BCL and major subtypes mutually adjusted for other PCs stratified by age
**FIGURE S1**. Heatmap of age‐, sex‐ and country‐adjusted Spearman correlation between each two variables
**FIGURE S2**. Circos plot showing Spearman correlation of exposures adjusted for age, sex, and country in the data
**FIGURE S3**. Cox regression for individual exposure adjusted for age and sex in total BCL and subtypes obtained from meta‐analysis of the country‐based estimates

**TABLE S7** Standard Cox regression for individual exposure adjusted for country stratified by age and sex in total BCL and subtypes

**TABLE S8** Component loading across the components and the total variance explained by each component from PCA analysis

## Data Availability

Data may not be shared as the EPIC explicitly retains ownership of the primary data. However, the EPIC data are available for external investigators who seek to answer important questions on health and disease in the context of research projects that are consistent with the legal and ethical standard practices of IARC/WHO and the EPIC Centres, and the analyses were conducted by (or in collaboration with) one or more EPIC investigators. The research protocol, data analysis plan, syntaxes and analysis files of current study are available on request.
