# Supplementary material for: Association between anthropometry and lifestyle factors and risk of B‐cell lymphoma: An exposome‐wide analysis
Source: Int J Cancer. 2020 Nov 12;148(9):2115–28. doi: 10.1002/ijc.33369 (PMC8048490; doi:10.1002/ijc.33369)
Supplement: Supplementary file 1 — TABLE S1 ICD‐O‐3 morphology codes TABLE S2. List of included exposures; mean and median for continuous exposures and frequency (%) of categorical exposures in total study population TABLE S3. Missing rate TABLE S4. Case number of BCL and major subtypes stratified by country TABLE S5. Univariate Cox model for individual exposure in total BCL and subtypes TABLE S6. Estimates and SE of the significant associated exposures from meta‐analysis of per‐country Cox model coefficients adjusted for age and gender (upper part) and estimates and SE of the significant associated exposures of Cox model adjusted for country, age, and sex (lower part) TABLE S9. HRs and 95%CI of the significant principal components for total BCL and major subtypes adjusted for age, gender, and country TABLE S10. Multivariate Cox regression of the principal components for total BCL and major subtypes mutually adjusted for other PCs stratified by age FIGURE S1. Heatmap of age‐, sex‐ and country‐adjusted Spearman correlation between each two variables FIGURE S2. Circos plot showing Spearman correlation of exposures adjusted for age, sex, and country in the data FIGURE S3. Cox regression for individual exposure adjusted for age and sex in total BCL and subtypes obtained from meta‐analysis of the country‐based estimates [file IJC-148-2115-s001.pdf]

Association between anthropometry and lifestyle factors and risk of B cell lymphoma: an exposome wide analysis

Fatemeh Saberi Hosnijeh, Delphine Casabonne, Alexandra Nieters, Marta Solans, Sabine Naudin, Pietro Ferrari, James D. Mckay, Yolanda Benavente, Elisabete Weiderpass, Heinz Freisling, Gianluca Severi, Marie-Christine Boutron Ruault, Caroline Besson, Claudia Agnoli, Giovanna Masala, Carlotta Sacerdote, Rosario Tumino, José María Huerta, Pilar Amiano, Miguel Rodriguez-Barranco, Catalina Bonet, Aurelio Barricarte, Sofia Christakoudi, Anika Knuppel, Bas Bueno-de-Mesquita, Matthias B. Schulze, Rudolf Kaaks, Federico Canzian, Florentin Späth, Mats Jerkeman, Charlotta Rylander, Anne Tjønneland, Anja Olsen, Kristin Benjaminsen Borch, Roel Vermeulen

Table of contents

Supplementary Table 1. ICD-O-3 morphology codes

Supplementary Table 2. List of included exposures; mean and median for continuous exposures and frequency (%) of categorical exposures in total study population

Supplementary Table 3. Missing rate

Supplementary Table 4. Case number of BCL and major subtypes stratified by country

Supplementary Table 5. Univariate Cox model for individual exposure in total BCL and subtypes

Supplementary Table 6. Estimates and SE of the significant associated exposures from meta-analysis of per-country Cox model coefficients adjusted for age and gender (upper part) and estimates and SE of the significant associated exposures of Cox model adjusted for country, age, and sex (lower part)

Supplementary Table 7 (Excel file). Standard Cox regression for individual exposure adjusted for country stratified by age and sex in total BCL and subtypes

Supplementary Table 8 (Excel file). Component loading across the components and the total variance explained by each component from PCA analysis

Supplementary Table 9. HRs and 95%CI of the significant principal components for total BCL and major subtypes adjusted for age, gender, and country

Supplementary Table 10. Multivariate Cox regression of the principal components for total BCL and major subtypes mutually adjusted for other PCs stratified by age

Supplementary Figure 1. Heatmap of age-, sex- and country- adjusted Spearman correlation between each two variables

Supplementary Figure 2. Circos plot showing Spearman correlation of exposures adjusted for age, sex, and country in the data

Supplementary Figure 3. Cox regression for individual exposure adjusted for age and sex in total BCL and subtypes obtained from meta-analysis of the country-based estimates

Supplementary Table 1. ICD-O-3 morphology codes

Code	BCL Subtype	Case Number
9670/3	B cell chronic lymphocytic leukemia/small lymphocytic leukemia	69
9823/3	B cell chronic lymphocytic leukemia/small lymphocytic leukemia	468
9679/3	Diffuse large B-cell lymphoma	1
9680/3	Diffuse large B-cell lymphoma	480
9684/3	Diffuse large B-cell lymphoma	7
9690/3	Follicular lymphoma	302
9691/3	Follicular lymphoma	29
9695/3	Follicular lymphoma	24
9698/3	Follicular lymphoma	26
9731/3	Plasma cell neoplasm/Multiple myeloma	49
9732/3	Plasma cell neoplasm/Multiple myeloma	615
9733/3	Plasma cell neoplasm/Multiple myeloma	5
9734/3	Plasma cell neoplasm/Multiple myeloma	7
9761/3	Lymphoplasmacytic Lymphoma	39
9671/3	Lymphoplasmacytic Lymphoma	62
9826/3	Burkitt cell leukemia	1
9687/3	Burkitt lymphoma	11
9832/3	Prolymphocytic leukemia, B- cell	4
9940/3	Hairy cell leukemia	31
9689/3	Marginal zone lymphoma	10
9699/3	Marginal zone lymphoma	90
9673/3	Mantle cell lymphoma	71
9678/3	Primary effusion lymphoma	1
Total		2402

Supplementary Table 2. List of included exposures; mean and median for continuous exposures and frequency (%) of categorical exposures in total study population

Category	Exposure	Remark	Mean	Median	%
Anthropometry	Body-mass index, (kg/m^2)	Continuous	25.4	24.8	
	Height (cm)	Continuous	166	165	
	Weight (kg)	Continuous	70.2	68.4	
	Hip circumference (cm)	Continuous	100.6	100	
	Waist circumference (cm)	Continuous	84.3	83.7	
	Waist to hip ratio	Continuous	0.84	0.83	
Smoking	Currently smoke cigarettes	Yes/no			22.2
	Currently smoke cigars	Yes/no			1.6
	Ever smoker	Yes/no			51
	Current smoker	Yes/no			23.6
	Duration of smoking (year)	Continuous	11.7	0	
	Duration of cigarettes smoking (year)	Continuous	11.1	0	
	Currently smoking >15/day	Yes/no			8.2
	Currently smoking >25/day	Yes/no			1.9
Alcohol	Alcohol at recruitment (g/day)	Continuous	11.6	5.3	
SEP	Education *	1-4			30.3, 23.8, 21, 24.9
Medical history	Myocardial infarction (MI)	Yes/no			1.4
	Stroke	Yes/no			2.6
	Hypertension	Yes/no			24.9
	Diabetes	Yes/no			2.8
	Cardiovascular problem reported	Yes/no			26.5
Physical activity	METS recreational activity	Continuous	31.4	27.0	
	METS household activity	Continuous	51.5	44.7	
	METS recreational and household activity	Continuous	82.9	78.0	
	Sedentary occupation	Yes/no			28.1
	Standing occupation	Yes/no			24.8
	Manual work	Yes/no			9.7
	Heavy manual work	Yes/no			2.7
	Total physical activity index (sex-specific) (Active)	Yes/no			10.7
	IARC physical activity score †	1-3			33.2, 33.4, 33.3
	IARC physical activity score (Intense)	Yes/no			33.3
	Cambridge physical activity index ‡	1-4			21, 33.8, 27, 18.2
	Cambridge physical activity index (Active)	Yes/no			18.2
Food group	Potatoes and other tubers (g/day)	Continuous	92.3	75.7	
	Vegetables (g/day)	Continuous	211.5	175.4	
	Legumes (g/day)	Continuous	14.6	5.7	
	Fruits, nuts and seeds (g/day)	Continuous	239.8	200.5	
	Dairy products (g/day)	Continuous	326.6	277.2	
	Cereal and cereal products (g/day)	Continuous	219.1	200.1	
	Meat and meat products (g/day)	Continuous	98.6	91.8	
	Fish and shellfish (g/day)	Continuous	37.1	28.0	
	Egg and egg products (g/day)	Continuous	17.7	14.0	
	Fat (g/day)	Continuous	28.2	25.3	
	Sugar and confectionary (g/day)	Continuous	41.7	30.7	

	Cakes and biscuits (g/day)	Continuous	40.8	29.4
	Non-alcoholic beverages (g/day)	Continuous	1060.3	994.8
	Alcoholic beverages (g/day)	Continuous	170.8	71.5
	Condiments and sauces (g/day)	Continuous	21.1	15.8
	Soups, bouillons (g/day)	Continuous	49.3	22.1
ENDB	Total proteins (g/day)	Continuous	86.7	83.6
	Carbohydrates (g/day)	Continuous	228.1	218.3
	Total fats (g/day)	Continuous	81.4	77.3
	Alcohol (g/day)	Continuous	12.0	5.7
	Cholesterol (mg/day)	Continuous	315.1	291.7
	Total saturated fatty acids (g/day)	Continuous	31.2	29.2
	Total monounsaturated fatty acids (g/day)	Continuous	30.0	27.4
	Total polyunsaturated fatty acids (g/day)	Continuous	13.4	12.2
	Total dietary fibre (g/day)	Continuous	22.8	21.8
	Vitamin C (mg/day)	Continuous	127.3	113.5
	Beta-carotene (µg/day)	Continuous	3577.7	2865.6
	Vitamin E (mg/day)	Continuous	12.2	10.9
	Potassium (mg/day)	Continuous	3678	3570
	Water (g/day)	Continuous	2219.2	2117.7
	Starch (g/day)	Continuous	120.9	113.3
	Calcium (mg/day)	Continuous	994.6	935.4
	Phosphorus (mg/day)	Continuous	1516.1	1454.6
	Iron (mg/day)	Continuous	13.1	12.5
	Sugar (g/day)	Continuous	102.9	96.0
	Magnesium (mg/day)	Continuous	359.9	345.3
	Retinol (µg/day)	Continuous	830.3	621.3
	Riboflavin, B2 (mg/day)	Continuous	1.86	1.73
	Thiamin, B1 (mg/day)	Continuous	1.34	1.25
	Cobalamin, B12 (µg/day)	Continuous	6.5	5.7
	Vitamin B6 (mg/day)	Continuous	1.88	1.79
	Vitamin D (µg/day)	Continuous	4.16	3.31
	Total proteins, animal origin (g/day)	Continuous	52.0	49.9
	Total proteins, plant origin (g/day)	Continuous	27.1	25.4
	Total fats, animal origin (g/day)	Continuous	36.5	33.7
	Total fats, plant origin (g/day)	Continuous	25.9	21.1
	Folate (µg/day)	Continuous	30.8.5	288.7
	Energy (kcal/day)	Continuous	2075	1996.9
Dietary pattern	Mediterranean diet score §	Continuous	7.8	8
	Inflammatory score of the diet	Continuous	0.257	0.342
	Inflammatory score of the diet-no alcohol §	Continuous	0.235	0.322

Socioeconomic position (SEP); * None/ Primary school, Technical/professional school, Secondary school, Longer education (incl. university degree); Nutrient density from EPIC Nutrient Data Base (ENDB), Metabolic Equivalent of Task/ week (METS); † Low, Medium, High; ‡ Inactive, moderately inactive, moderately active, active; § excluding alcohol

Supplementary Table 3. Missing rate

Variables	Missing rate %
Hip circumference	22.2
Waist circumference	22.2
Education	3.6
Myocardial infarction	3.2
Stroke	8.8
Hypertension	10.9
Diabetes	3.5
Cardiovascular problem reported	15.4
METS recreational activity	13.7
METS household activity	13.7
METS recreational and household activity	13.7
PA-Work	10.1
Total physical activity index	6.9
IARC physical activity score	1.4
Cambridge physical activity index	1.9
Currently smoke cigarette	1.5
Currently smoke cigars	1.4
Smoking status	2.0
Duration of smoking	4.3
Duration of cigarette smoking	2.5
Smoke Intensity	1.7

*PA: physical activity; Metabolic Equivalent of Task/ week (METS)

Supplementary Table 4. Case number of BCL and major subtypes stratified by country

Country	BCL, n=2402	DLBCL, n=488	CLL, n=537	FL, n=381	MM, n=676
France	205 (8.5%)	40 (8.2%)	44 (8.2%)	44 (11.5%)	45 (6.7%)
Italy	218 (9.1%)	38 (7.8%)	44 (8.2%)	33 (8.7%)	73 (10.8%)
Spain	194 (8.1%)	35 (7.2%)	51 (9.5%)	27 (7.1%)	51 (7.5%)
UK	426 (17.7%)	95 (19.5%)	87 (16.2%)	71 (18.6%)	115 (17.0%)
Netherlands	172 (7.2%)	43 (8.8%)	41 (7.6%)	26 (6.8%)	43 (6.4%)
Greece	38 (1.6%)	3 (0.6%)	13 (2.4%)	3 (0.8%)	15 (2.2%)
Germany	170 (7.1%)	30 (6.1%)	39 (7.3%)	20 (5.2%)	55 (8.1%)
Sweden	344 (14.3%)	57 (11.7%)	74 (13. 8%)	48 (12.6%)	132 (19.5%)
Denmark	506 (21.1%)	121 (24.8%)	118 (22.0%)	78 (20.5%)	123 (18.2%)
Norway	129 (5.4%)	26 (5.3 %)	26 (4.8%)	31 (8.1%)	24 (3.6%)

Supplementary Table 5. Univariate Cox model for individual exposure in total BCL and subtypes (black: significant after FDR multiple testing correction)

	BCL			CLL			FL			DLBCL			MM		
Exposure	Estimate	SE	pFDR	Estimate	SE	pFDR	Estimate	SE	pFDR	Estimate	SE	pFDR	Estimate	SE	pFDR
Height	0.02187	0.00221	0	0.02912	0.00463	6.9E-09	0.01577	0.00558	0.08883	0.02125	0.00489	0.00014	0.02041	0.00417	9.3E-06
Weight	0.01638	0.00138	0	0.01885	0.00287	1.4E-09	0.00561	0.00369	0.42824	0.01806	0.00304	1.2E-07	0.02152	0.00252	0
BMI	0.03136	0.00449	2.3E-11	0.03194	0.00947	0.00338	-0.00087	0.01218	0.95371	0.03856	0.00979	0.00054	0.05147	0.00799	2.5E-09
Hip	0.01381	0.00246	9.7E-08	0.01621	0.00515	0.00646	0.00512	0.00642	0.62879	0.01527	0.00544	0.01737	0.02060	0.00450	4.1E-05
Waist	0.02044	0.00160	0	0.02554	0.00332	6.1E-13	0.00517	0.00422	0.52486	0.01936	0.00357	1.3E-06	0.02591	0.00296	0
WHR	2.73127	0.21009	0	3.42852	0.42835	1.1E-13	0.57214	0.56234	0.5810	2.47944	0.47198	2.6E-06	3.23438	0.38712	3.2E-15
Alcohol at recruitment	0.00587	0.00106	1.2E-07	0.00877	0.00203	0.00014	0.00433	0.00278	0.41571	0.00488	0.00243	0.09165	0.00351	0.00214	0.16522
Potatoes, tubers	0.00172	0.00022	5.9E-14	0.00192	0.00046	0.00020	0.00075	0.00063	0.52486	0.00195	0.00046	0.00024	0.00212	0.00039	9.1E-07
Vegetables	-0.00066	0.00016	5.4E-05	-0.00070	0.00033	0.07782	-0.00035	0.00038	0.5935	-0.00087	0.00035	0.03986	-0.00075	0.00030	0.03376
Legumes	-0.00641	0.00104	3.6E-09	-0.00578	0.00217	0.02514	-0.00598	0.00259	0.16649	-0.00415	0.00215	0.10772	-0.00861	0.00209	0.00028
Fruits, nuts, seeds	-0.00044	0.00012	0.00052	-0.00031	0.00025	0.33256	-0.00032	0.00029	0.57176	-0.00043	0.00026	0.18216	-0.00041	0.00022	0.13893
Dairy products	0.00037	0.00008	1.0E-05	0.00032	0.00017	0.13220	0.00044	0.00020	0.18644	0.00066	0.00016	0.00042	0.00032	0.00015	0.07502
Cereal	-0.00002	0.00018	0.93372	-0.00023	0.00039	0.64779	-0.00019	0.00046	0.87537	-0.00056	0.00042	0.28408	0.00039	0.00033	0.33636
Meat	0.00172	0.00032	2.4E-07	0.00283	0.00064	0.00011	-0.00013	0.00085	0.91862	0.00135	0.00071	0.11584	0.00181	0.00060	0.01183
Fish and shellfish	0.00127	0.00054	0.02754	0.00208	0.00108	0.11784	0.00259	0.00124	0.22992	-0.00001	0.00127	0.99743	0.00032	0.00106	0.80305
Egg	0.00222	0.00113	0.07186	0.00655	0.00196	0.00366	-0.00129	0.00312	0.87537	0.00301	0.00246	0.31035	-0.00147	0.00235	0.62003
Fat	0.00045	0.00111	0.72694	0.00035	0.00236	0.91220	-0.00823	0.00311	0.09992	-0.00164	0.00252	0.63061	0.00567	0.00193	0.01479
Sugar, confectionary	0.00182	0.00025	8.5E-12	0.00119	0.00068	0.14653	0.00260	0.00045	5.3E-07	0.00197	0.00053	0.00113	0.00150	0.00054	0.01995
Cakes, biscuits	0.00072	0.00047	0.16494	0.00013	0.00103	0.92385	-0.00035	0.00125	0.89907	0.00012	0.00108	0.98896	0.00179	0.00082	0.07262
Non-Alcoholic beverages	0.00016	0.00003	2.4E-09	0.00014	0.00005	0.03450	0.00016	0.00006	0.11414	0.00030	0.00005	4.1E-07	0.00010	0.00005	0.07502
Alcoholic beverages	0.00039	0.00006	1.3E-10	0.00051	0.00011	3.1E-05	0.00031	0.00016	0.28379	0.00034	0.00013	0.03506	0.00029	0.00012	0.04171
Condiments, sauces	-0.00299	0.00105	0.00755	-0.00631	0.00240	0.02595	0.00118	0.00239	0.87180	-0.00498	0.00242	0.08711	-0.00291	0.00198	0.21436
Soups, bouillons	-0.00028	0.00029	0.36900	-0.00020	0.00060	0.81110	-0.00222	0.00086	0.10468	-0.00083	0.00068	0.31035	-0.00001	0.00053	0.98301
Protein	0.00283	0.00072	0.00018	0.00400	0.00150	0.02514	0.00189	0.00182	0.58000	0.00205	0.00161	0.30269	0.00214	0.00137	0.18924
Carbohydrate	0.00103	0.00027	0.00022	0.00055	0.00057	0.48747	0.00118	0.00066	0.38471	0.00083	0.00059	0.2555	0.00141	0.00049	0.01726
Fat	0.00110	0.00068	0.14249	0.00054	0.00144	0.78792	-0.00075	0.00174	0.87537	-0.00040	0.00153	0.90555	0.00186	0.00126	0.21436

Alcohol	0.00562	0.00105	3.1E-07	0.00864	0.00200	0.00014	0.00456	0.00272	0.39592	0.00416	0.00243	0.15533	0.00323	0.00212	0.19621
Cholestrol	0.00084	0.00012	9E-11	0.00112	0.00025	0.00011	0.00046	0.00032	0.48812	0.00080	0.00028	0.01352	0.00069	0.00024	0.01479
Saturated FA	0.00649	0.00150	4.4E-05	0.00464	0.00322	0.26071	0.00358	0.00386	0.59356	0.00481	0.00337	0.2555	0.00788	0.00280	0.01852
Monounsaturated FA	-0.00087	0.00155	0.62654	-0.00136	0.00328	0.77232	-0.00592	0.00406	0.46698	-0.00651	0.00361	0.13473	0.00157	0.00286	0.65782
Polyunsaturated FA	-0.00459	0.00338	0.21772	-0.00859	0.00728	0.37042	-0.01147	0.00877	0.48812	-0.00949	0.00767	0.31035	0.00013	0.00624	0.98301
Dietary fibre	0.00341	0.00257	0.22527	0.00505	0.00541	0.50012	-0.00185	0.00659	0.89907	0.00649	0.00561	0.34167	0.00301	0.00485	0.62003
Vit C	-0.00151	0.00033	1.2E-05	-0.00122	0.00068	0.13872	-0.00136	0.00081	0.39592	-0.00189	0.00074	0.03185	-0.00149	0.00061	0.04171
Beta carotene	-0.00001	0.00001	0.24203	-0.00001	0.00002	0.51031	0.00001	0.00002	0.59356	0.00000	0.00002	0.96427	-0.00002	0.00001	0.25014
Vit E	-0.01943	0.00391	2.3E-06	-0.02551	0.00844	0.00909	-0.02751	0.01013	0.0962	-0.02954	0.00905	0.00530	-0.01264	0.00718	0.14033
Potassium	0.00006	0.00002	0.00135	0.00007	0.00004	0.13872	0.00007	0.00005	0.48812	0.00013	0.00004	0.00727	0.00003	0.00004	0.50853
Water	0.00016	0.00002	2.9E-13	0.00017	0.00005	0.00088	0.00015	0.00005	0.08883	0.00027	0.00005	1.2E-07	0.00012	0.00004	0.01726
Starch	0.00062	0.00040	0.16494	0.00028	0.00087	0.81110	-0.00030	0.00104	0.89907	-0.00093	0.00094	0.42214	0.00191	0.00073	0.02937
Calcium	0.00015	0.00005	0.00358	0.00009	0.00010	0.51031	0.00019	0.00012	0.41571	0.00023	0.00010	0.05248	0.00010	0.00009	0.34426
Phosphorus	0.00019	0.00004	1.2E-05	0.00024	0.00009	0.01932	0.00014	0.00010	0.48812	0.00022	0.00009	0.03819	0.00015	0.00008	0.12451
Iron	0.00372	0.00473	0.48151	0.01212	0.00982	0.34323	-0.01043	0.01224	0.62339	-0.00475	0.01066	0.76098	0.00266	0.00893	0.80305
Sugar	0.00184	0.00045	9.8E-05	0.00081	0.00097	0.52030	0.00315	0.00108	0.08883	0.00287	0.00096	0.01185	0.00150	0.00085	0.14033
Magnesium	0.00040	0.00018	0.03922	0.00027	0.00039	0.60528	0.00037	0.00046	0.62879	0.00082	0.00039	0.08615	0.00014	0.00035	0.74782
Retinol	0.00008	0.00002	0.00028	0.00006	0.00005	0.39948	0.00008	0.00005	0.41571	0.00010	0.00004	0.02183	0.00009	0.00004	0.04329
Riboflavin	0.09140	0.02583	0.00079	0.09505	0.05445	0.14653	0.08348	0.06510	0.49654	0.16410	0.05476	0.01185	0.04246	0.05007	0.49271
Thiamin	0.06059	0.03979	0.16603	0.07230	0.08398	0.51679	-0.02525	0.10285	0.89907	0.14738	0.08531	0.15235	0.01054	0.07634	0.92197
Vit B12	0.01746	0.00440	0.00017	0.02111	0.00896	0.05029	0.02379	0.01031	0.16649	0.01423	0.01005	0.2555	0.01427	0.00856	0.16305
Vit B6	0.11690	0.03184	0.00051	0.15384	0.06664	0.05366	0.08723	0.08066	0.57176	0.09962	0.07096	0.2555	0.11042	0.06013	0.13412
Vit D	0.02175	0.00511	5.3E-05	0.02670	0.01017	0.02595	0.02143	0.01286	0.39592	-0.00759	0.01414	0.70475	0.02933	0.00882	0.00480
Protein-animal	0.00450	0.00086	6.7E-07	0.00682	0.00178	0.00075	0.00297	0.00220	0.48812	0.00390	0.00192	0.09165	0.00328	0.00165	0.10403
Protein-plant	-0.00293	0.00197	0.17284	-0.00358	0.00419	0.51679	-0.00197	0.00492	0.87537	-0.00419	0.00440	0.44132	-0.00435	0.00375	0.33757
Fat-animal	0.00679	0.00106	7.2E-10	0.00788	0.00221	0.00171	0.00251	0.00280	0.59356	0.00609	0.00236	0.03185	0.00641	0.00200	0.00702
Fat-plant	-0.00989	0.00134	1.49E-12	-0.01160	0.00288	0.00037	-0.01180	0.00344	0.02662	-0.01511	0.00317	2.3E-05	-0.00620	0.00241	0.03128
Energy	0.00014	0.00003	4.85E-05	0.00013	0.00007	0.11784	0.00009	0.00008	0.52554	0.00008	0.00007	0.36166	0.00015	0.00006	0.03128
Folate	-0.00026	0.00017	0.17284	-0.00023	0.00036	0.62962	-0.00015	0.00043	0.89647	-0.00003	0.00037	0.98896	-0.00064	0.00034	0.12451
MED_score	-0.05833	0.00680	0	-0.05496	0.01437	0.00075	-0.02647	0.01698	0.41571	-0.07605	0.01514	7.4E-06	-0.06738	0.01285	1.9E-06

ISD	0.00468	0.02030	0.84966	-0.01055	0.04285	0.85466	-0.00250	0.05093	0.96077	0.00093	0.04497	0.99743	0.02546	0.03842	0.60474
ISD-no alcohol	0.00989	0.02032	0.67280	-0.00317	0.04291	0.95208	0.00424	0.05099	0.95371	0.00059	0.04497	0.99743	0.03134	0.03847	0.50853
MI	0.43936	0.15050	0.00610	0.60927	0.29199	0.08237	-0.84147	0.70899	0.52486	0.95435	0.26231	0.00159	0.29318	0.30402	0.42223
Stroke	0.02871	0.12642	0.84966	0.04226	0.26535	0.91220	-0.10001	0.33820	0.89907	-0.37190	0.33730	0.36166	0.36257	0.20428	0.14033
Hypertension	0.16478	0.04558	0.00060	0.21041	0.09560	0.06896	-0.05622	0.12076	0.87537	0.07072	0.10309	0.61234	0.43863	0.08157	1.1E-06
Diabetes	0.27044	0.11585	0.02951	0.51036	0.21776	0.05034	-0.16087	0.35736	0.87537	0.16907	0.27121	0.64407	0.53479	0.19306	0.01995
CVD	0.22350	0.04445	1.7E-06	0.30437	0.09258	0.00418	-0.02720	0.11809	0.90061	0.28303	0.09750	0.01352	0.36289	0.08167	7.0E-05
METS household activity	-0.00190	0.00055	0.00105	-0.00287	0.00119	0.04348	-0.00242	0.00139	0.39592	-0.00151	0.00121	0.30992	-0.00114	0.00102	0.34426
METS recreational activity	0.00092	0.00084	0.31942	0.00047	0.00179	0.84933	0.00057	0.00212	0.89907	0.00394	0.00175	0.06054	-0.00068	0.00163	0.74782
METS household/ recreational	-0.00105	0.00045	0.02951	-0.00183	0.00097	0.12109	-0.00150	0.00114	0.48812	0.00010	0.00097	0.98896	-0.00098	0.00085	0.33757
Total PA Sx (Active)	0.08068	0.06255	0.23819	-0.05712	0.14014	0.77232	0.26405	0.14694	0.38471	0.29706	0.12770	0.05248	0.01058	0.12110	0.95229
Sedentary occupation	-0.13943	0.04673	0.00505	-0.06937	0.09720	0.59088	0.07487	0.11189	0.72993	-0.21987	0.10573	0.08615	-0.31152	0.09213	0.00430
Standing occupation	-0.28185	0.05076	1.2E-07	-0.42631	0.11220	0.00078	-0.04527	0.11929	0.87537	-0.49707	0.12051	0.00029	-0.23736	0.09443	0.03466
Manual work	0.18032	0.06258	0.0066	0.14058	0.13512	0.43964	0.20565	0.15589	0.48812	0.37594	0.12851	0.01352	0.18223	0.11789	0.19324
Heavy manual work	0.07901	0.11809	0.55440	0.38136	0.21772	0.14653	-0.19455	0.33735	0.80459	0.00087	0.27119	0.99743	0.08829	0.22170	0.75087
Cambridge PA	-0.00187	0.02011	0.93372	0.02770	0.04250	0.62180	0.05515	0.05039	0.57176	0.02037	0.04450	0.76078	-0.04786	0.03804	0.30204
Cambridge PA (Active)	0.09018	0.05080	0.10477	0.22395	0.10362	0.07414	0.10633	0.12700	0.62518	0.14861	0.11058	0.27802	0.05663	0.09677	0.63920
IARC PA	0.07288	0.02506	0.0062	0.04531	0.05296	0.51679	0.01614	0.06283	0.89907	0.13223	0.05577	0.04820	0.05634	0.04721	0.33191
IARC PA (Intense)	0.10212	0.04259	0.02611	0.05797	0.09071	0.62304	0.01013	0.10847	0.95371	0.20643	0.09321	0.06471	0.08781	0.08045	0.35719
Currently smoke cigarette	-0.13060	0.05131	0.01759	-0.16307	0.10961	0.24291	-0.12977	0.12885	0.58101	-0.10672	0.11300	0.44132	-0.16103	0.09759	0.16522
Currently smoke cigars	-0.15341	0.17273	0.42308	0.01931	0.33618	0.95420	-0.45307	0.50266	0.59356	-0.00785	0.35651	0.99743	-0.10981	0.31861	0.78445
Ever smoker	0.15659	0.04096	0.00028	0.18391	0.08674	0.07782	-0.01743	0.10247	0.91862	0.16807	0.09091	0.12471	0.16998	0.07725	0.06906
Current smoker	-0.10779	0.04986	0.0444	-0.08863	0.10487	0.51679	-0.12338	0.12579	0.59208	-0.08116	0.10979	0.57970	-0.16091	0.09542	0.15964
Smoking duration	0.00992	0.00132	5.9E-13	0.01330	0.00273	1.9E-05	0.00055	0.00352	0.91862	0.01250	0.00288	0.00014	0.00993	0.00249	0.00043
Cigarette smoking duration	0.00885	0.00137	6.4E-10	0.01170	0.00286	0.00030	0.00066	0.00362	0.91862	0.01087	0.00301	0.00165	0.00873	0.00259	0.00430
Currently smoking >15/day	-0.15371	0.08160	0.08362	-0.18975	0.17484	0.41667	-0.21972	0.21089	0.58000	-0.01564	0.17103	0.98896	-0.28836	0.16302	0.14033
Currently smoking >25/day	0.02388	0.15755	0.90024	0.36283	0.28082	0.32850	-0.46781	0.50269	0.59356	0.49528	0.28119	0.14470	-0.35003	0.35570	0.41592
Education	-0.11965	0.01792	1.5E-10	-0.11527	0.03784	0.00876	-0.03532	0.04442	0.62879	-0.13106	0.03987	0.00517	-0.17386	0.03418	3.9E-06

Supplementary Table 6. Estimates and SE of the significant (P<0.05) associated exposures from meta-analysis of per-country Cox model coefficients adjusted for age and gender (upper part) and estimates and SE of the significant associated exposures of Cox model adjusted for country, age, and sex (lower part)

	BCL		CLL		FL		DLBCL		MM	
**Exposure**	**Estimate**	**SE**	**Estimate**	**SE**	**Estimate**	**SE**	**Estimate**	**SE**	**Estimate**	**SE**
Height	**0.014**	**0.003**	0.019	0.008	0.020	0.008	0.016	0.007		
Weight	0.006	0.002					**0.013**	**0.003**	**0.013**	**0.003**
BMI							0.030	0.011	**0.032**	**0.009**
Hip circumference	0.010	0.003					0.012	0.006	**0.017**	**0.005**
Waist circumference	0.005	0.002							0.011	0.004
Dairy products	0.0002	0.0001								
Total saturated fatty acids	0.004	0.002								
Total fats, animal origin	0.003	0.001								
Sugar and confectionary					0.004	0.001				
Sugar					0.003	0.001				
Condiments and sauces	-0.002	0.001								
Egg and egg products			0.005	0.002			0.005	0.002		
Vit D							-0.041	0.018		
METS recreational activity							0.004	0.002		
Total PA (sex-specific) (Active)	0.146	0.064			0.370	0.152	0.388	0.132		
Manual work	0.166	0.064					**0.419**	**0.135**	0.248	0.122
Diabetes			0.498	0.225					0.325	0.367
MI							0.606	0.266		
Stroke									0.505	0.212
Currently smoking >25/day			0.868	0.287	1.212	0.549	**0.928**	**0.284**		
Height	**0.014**	**0.003**	0.018	0.007	0.020	0.008	0.016	0.007		
Weight	**0.007**	**0.002**					0.012	0.004	**0.012**	**0.003**
BMI	0.011	0.005					0.025	0.011	**0.031**	**0.009**
Hip circumference	**0.010**	**0.003**					0.012	0.006	**0.017**	**0.005**
Waist circumference	0.005	0.002							0.010	0.004

Total fats, animal origin	0.003	0.001						
Total saturated fatty acids	0.003	0.002						
Condiments and sauces	-0.002	0.001	-0.005	0.003				
Total fats, plant origin			-0.008	0.004				
Sugar and confectionary					**0.002**	**0.001**		
Sugar					0.002	0.001		
Dairy products	0.0002	0.025					0.0004	0.0002
Vit D							-0.043	0.018
Manual work	0.156	0.064					0.354	0.132
Total PA (sex-specific) (Active)	0.130	0.064			0.307	0.150	0.355	0.130
Current smoker	-0.108	0.051						
Currently smoking >25/day							0.706	0.283

Bold: significant after FDR multiple testing correction

Supplementary Table 7 (Excel file). Standard Cox regression for individual exposure adjusted for country stratified by age and sex in total BCL and subtypes

Supplementary Table 8 (Excel file). Component loading across the components and the total variance explained by each component from PCA analysis

Supplementary Table 9. HRs and 95%CI of the significant ($p<0.05$) principal components for total BCL and major subtypes adjusted for age, gender, and country

			BCL		CLL		FL		DLBCL		MM	
**PC**	**Exposure**	**Loading**	**HR (95% CI)**	**P**	**HR (95% CI)**	**P**	**HR (95% CI)**	**P**	**HR (95% CI)**	**P**	**HR (95% CI)**	**P**
5	Weight	0.915	**1.08 (1.03-1.13)**	**0.001**					1.13 (1.03-1.25)	0.01	**1.16 (1.07-1.26)**	**0.0003**
	Waist circumference	0.901										
	Body Mass Index	0.895										
	Hip circumference	0.822										
	WHR	0.596										
7	Dairy products	0.896	1.05 (1.01-1.09)	0.02					1.09 (1.01-1.19)	0.04		
	Calcium (mg)	0.806										
	Riboflavin, B2 (mg)	0.626										
	Phosphorus (mg)	0.546										
10	Cambridge physical activity index	0.821	1.04 (1.01-1.08)	0.04					1.10 (1.01-1.20)	0.04		
	Cambridge physical activity index (Active)	0.813										
	Manual work	0.686										
	Total PA (sex-specific) (Active)	0.659										
11	Sugar (g)	0.741					1.11 (1.02-1.21)	0.02				
	Sugar and confectionary	0.662										
	Cakes and biscuits	0.635										
	Carbohydrates (g)	0.546										
12	Non-alcoholic beverages	0.848									1.13 (1.01-1.25)	0.03
	Water (g)	0.710										
15	Fish and shellfish	0.797							0.84 (0.75-0.95)	0.004		
	Vitamin D (µg)	0.771										

Bold: significant after FDR multiple testing correction

Supplementary Table 10. Multivariate Cox regression of the principal components for total BCL and major subtypes mutually adjusted for other PCs stratified by age

	HR (95% CI)									
	**BCL**		**CLL**		**FL**		**DLBCL**		**MM**	
**PC**	**Age≤55**	**Age>55**	**Age≤55**	**Age>55**	**Age≤55**	**Age>55**	**Age≤55**	**Age>55**	**Age≤55**	**Age>55**
1	0.95 (0.88-1.03)	0.93 (0.87-1.01)	0.97 (0.82-1.16)	0.92 (0.79-1.07)	0.97 (0.82-1.15)	0.95 (0.76-1.18)	1.02 (0.87-1.21)	0.92 (0.78-1.09)	0.90 (0.76-1.05)	0.91 (0.79-1.04)
2	0.97 (0.91-1.05)	1.06 (1.00-1.14)	1.00 (0.85-1.18)	**1.15 (1.01-1.31)**	0.92 (0.78-1.09)	1.03 (0.84-1.27)	0.94 (0.80-1.10)	1.01 (0.87-1.18)	1.03 (0.89-1.19)	0.98 (0.86-1.10)
3	0.96 (0.90-1.02)	1.00 (0.95-1.06)	0.90 (0.78-1.05)	1.01 (0.90-1.13)	0.98 (0.85-1.12)	0.90 (0.75-1.08)	0.89 (0.77-1.03)	1.10 (0.97-1.24)	1.06 (0.94-1.21)	0.94 (0.84-1.04)
4	1.03 (0.97-1.10)	1.05 (0.98-1.12)	1.07 (0.93-1.24)	0.96 (0.84-1.10)	1.01 (0.87-1.18)	1.06 (0.88-1.28)	0.95 (0.82-1.11)	1.03 (0.89-1.19)	1.09 (0.96-1.23)	1.06 (0.95-1.19)
5	**1.11 (1.03-1.20)**	1.06 (0.99-1.13)	**1.20 (1.02-1.41)**	0.96 (0.84-1.10)	1.06 (0.90-1.26)	0.94 (0.76-1.15)	1.12 (0.95-1.31)	1.16 (1.00-1.34)	1.12 (0.97-1.30)	**1.18 (1.06-1.33)**
6	0.99 (0.92-1.07)	1.02 (0.95-1.09)	0.88 (0.74-1.05)	0.94 (0.81-1.09)	1.00 (0.84-1.18)	0.89 (0.71-1.12)	0.88 (0.74-1.06)	1.02 (0.87-1.20)	1.10 (0.96-1.27)	1.03 (0.91-1.17)
7	**1.06 (1.00-1.13)**	1.03 (0.98-1.09)	1.07 (0.93-1.22)	0.97 (0.86-1.08)	1.11 (0.98-1.26)	1.05 (0.89-1.24)	1.09 (0.96-1.24)	**1.15 (1.02-1.29)**	1.02 (0.90-1.16)	1.02 (0.92-1.13)
8	0.96 (0.90-1.03)	1.04 (0.98-1.10)	0.97 (0.84-1.13)	0.98 (0.87-1.10)	0.91 (0.78-1.05)	1.03 (0.88-1.22)	1.01 (0.88-1.17)	1.06 (0.94-1.20)	0.93 (0.81-1.06)	1.05 (0.95-1.16)
9	1.02 (0.96-1.09)	1.02 (0.97-1.08)	1.01 (0.88-1.16)	1.06 (0.96-1.18)	1.12 (0.99-1.27)	0.95 (0.78-1.16)	1.03 (0.91-1.17)	1.00 (0.87-1.15)	0.97 (0.85-1.10)	0.98 (0.87-1.09)
10	1.02 (0.96-1.08)	**1.09 (1.03-1.16)**	1.05 (0.91-1.21)	1.08 (0.96-1.22)	1.07 (0.93-1.22)	1.09 (0.91-1.31)	1.04 (0.91-1.19)	**1.17 (1.03-1.33)**	1.02 (0.90-1.16)	1.08 (0.97-1.20)
11	1.04 (0.98-1.11)	1.04 (0.98-1.10)	1.03 (0.90-1.19)	0.95 (0.85-1.07)	1.11 (0.98-1.26)	**1.18 (1.01-1.38)**	1.06 (0.94-1.20)	1.06 (0.93-1.20)	1.02 (0.90-1.15)	1.02 (0.93-1.13)
12	1.01 (0.91-1.12)	1.09 (0.99-1.20)	1.07 (0.86-1.33)	1.06 (0.87-1.28)	1.00 (0.81-1.25)	1.00 (0.75-1.33)	1.00 (0.80-1.25)	1.17 (0.96-1.44)	1.11 (0.91-1.36)	1.08 (0.91-1.27)
13	1.01 (0.92-1.10)	1.05 (0.97-1.14)	1.02 (0.84-1.24)	0.98 (0.83-1.15)	1.02 (0.84-1.24)	1.02 (0.80-1.31)	1.02 (0.84-1.24)	**1.20 (1.01-1.43)**	1.01 (0.85-1.20)	1.09 (0.95-1.26)
14	**0.92 (0.86-0.99)**	1.01 (0.95-1.07)	0.96 (0.83-1.11)	0.99 (0.89-1.11)	0.88 (0.75-1.03)	0.95 (0.81-1.13)	0.92 (0.80-1.07)	1.04 (0.93-1.18)	0.95 (0.83-1.12)	1.06 (0.96-1.17)
15	1.00 (0.93-1.08)	0.98 (0.91-1.06)	1.11 (0.96-1.29)	0.98 (0.84-1.14)	1.10 (0.95-1.27)	0.98 (0.78-1.25)	**0.77 (0.64-0.93)**	0.97 (0.81-1.15)	1.01 (0.86-1.17)	1.08 (0.95-1.22)
16	0.97 (0.90-1.05)	1.06 (0.99-1.14)	1.06 (0.90-1.25)	1.04 (0.90-1.20)	1.03 (0.87-1.23)	1.15 (0.93-1.43)	0.89 (0.75-1.05)	1.15 (0.98-1.35)	0.96 (0.82-1.12)	0.98 (0.86-1.11)
17	0.95 (0.88-1.03)	0.95 (0.88-1.02)	0.97 (0.81-1.17)	0.87 (0.75-1.03)	0.98 (0.83-1.17)	0.89 (0.70-1.13)	1.06 (0.90-1.26)	1.00 (0.88-1.19)	**0.83 (0.70-0.98)**	0.97 (0.85-1.12)
18	1.01 (0.95-1.07)	0.99 (0.92-1.06)	1.06 (0.94-1.20)	0.97 (0.84-1.12)	0.99 (0.86-1.14)	1.00 (0.79-1.26)	1.07 (0.96-1.21)	1.02 (0.88-1.19)	0.93 (0.81-1.06)	0.94 (0.82-1.09)
19	0.99 (0.93-1.06)	1.00 (0.94-1.07)	0.91 (0.78-1.07)	0.99 (0.87-1.11)	0.99 (0.85-1.15)	1.10 (0.94-1.29)	0.99 (0.85-1.15)	1.04 (0.90-1.20)	0.99 (0.86-1.14)	1.00 (0.90-1.12)
20	0.98 (0.92-1.04)	0.98 (0.91-1.05)	0.91 (0.80-1.04)	0.94 (0.82-1.09)	1.01 (0.89-1.14)	0.88 (0.71-1.09)	0.88 (0.76-1.01)	1.00 (0.86-1.17)	1.07 (0.95-1.20)	0.99 (0.87-1.12)
21	0.97 (0.90-1.05)	1.03 (0.98-1.08)	0.99 (0.84-1.17)	1.03 (0.94-1.13)	0.96 (0.81-1.15)	0.87 (0.72-1.06)	1.03 (0.88-1.21)	1.06 (0.96-1.17)	0.89 (0.75-1.07)	1.06 (0.98-1.14)
22	0.98 (0.93-1.04)	0.97 (0.92-1.03)	1.06 (0.96-1.17)	1.01 (0.91-1.12)	0.91 (0.80-1.05)	0.98 (0.82-1.16)	0.98 (0.89-1.09)	0.89 (0.76-1.03)	0.92 (0.81-1.04)	1.04 (0.95-1.14)

Model included sex, country, age, and all PCs. Bold: significant at $p<0.05$

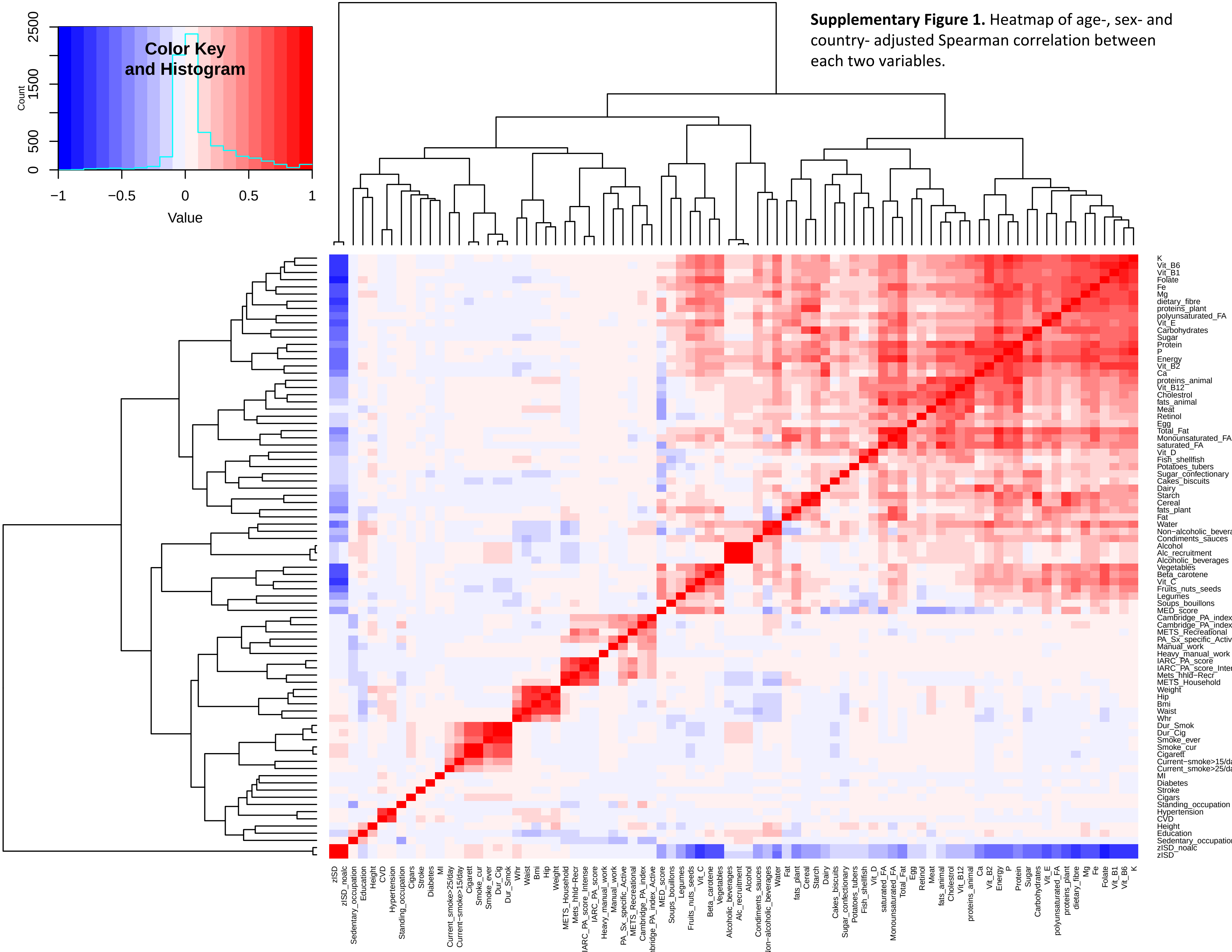

Supplementary Figure 1. Heatmap of age-, sex- and country- adjusted Spearman correlation between each two variables.

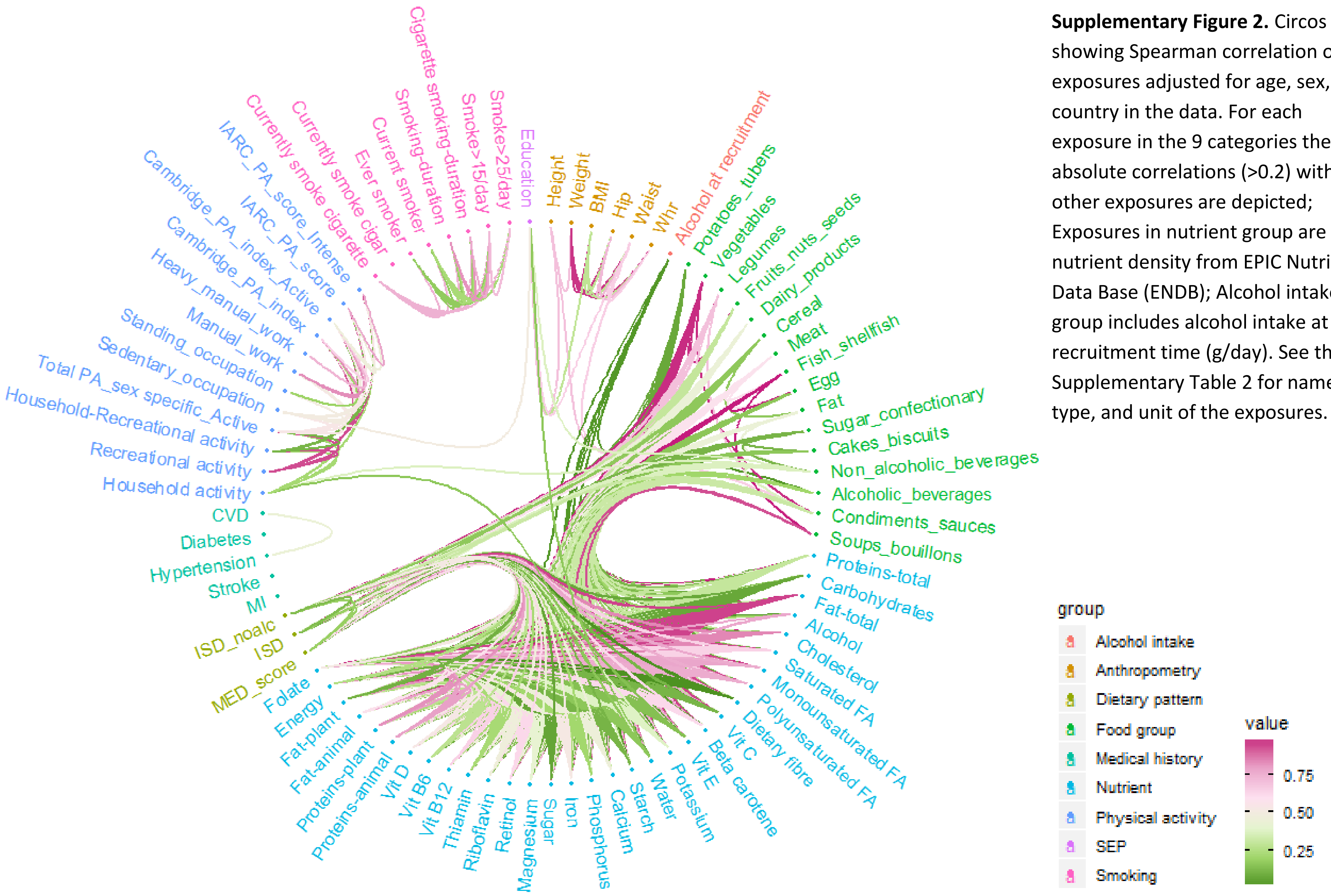

Supplementary Figure 2. Circos plot showing Spearman correlation of exposures adjusted for age, sex, and country in the data. For each exposure in the 9 categories the absolute correlations (>0.2) with all other exposures are depicted; Exposures in nutrient group are nutrient density from EPIC Nutrient Data Base (ENDB); Alcohol intake group includes alcohol intake at recruitment time (g/day). See the Supplementary Table 2 for name, type, and unit of the exposures.

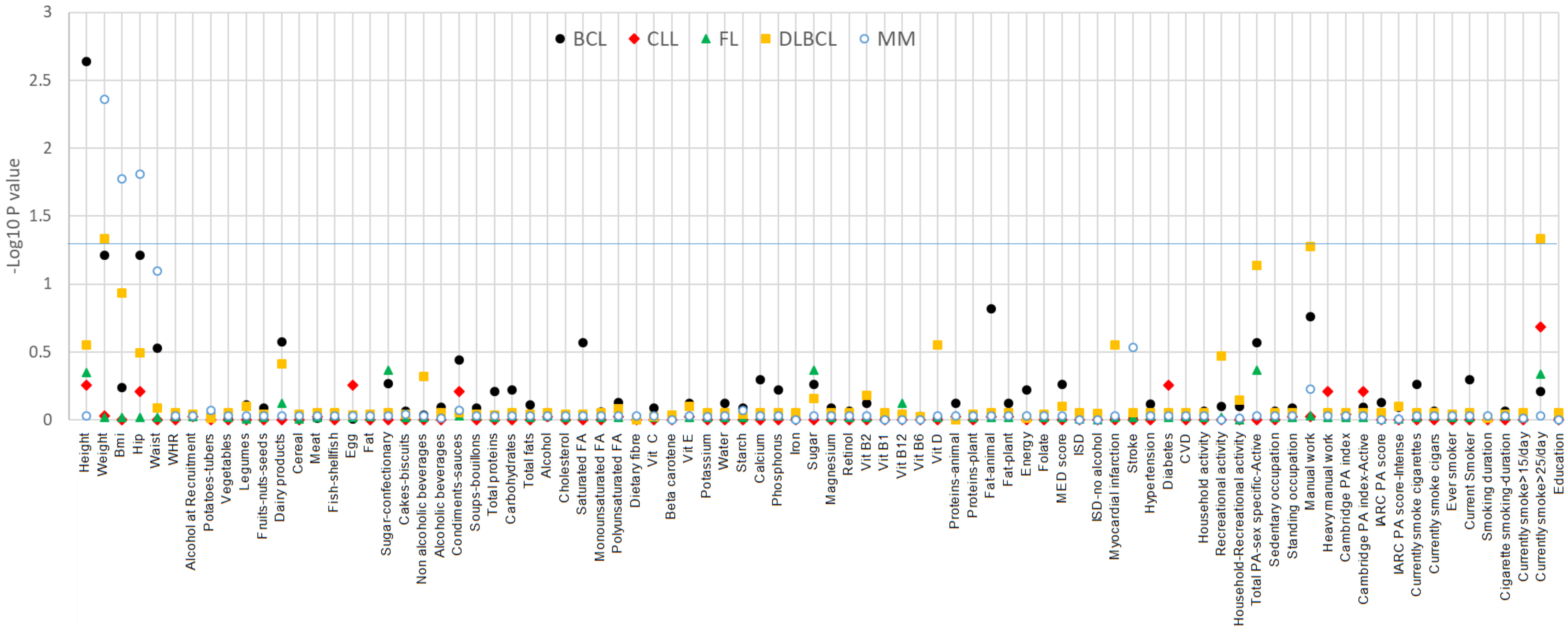

Supplementary Figure 3. Cox regression for individual exposure adjusted for age and sex in total BCL and subtypes obtained from meta-analysis of the country-based estimates. Vertical axis shows the –Log10 pFDR and blue line shows pFDR=0.05
